# Deep Learning-Based Denoising of Noisy Vibration Signals from Wavefront Sensors Using BiL-DCAE

**DOI:** 10.3390/s25165012

**Published:** 2025-08-13

**Authors:** Yun Pan, Quan Luo, Yiyou Fan, Haoming Chen, Donghua Zhou, Hongsheng Luo, Wei Jiang, Jinshan Su

**Affiliations:** 1 Key Laboratory of Vibration Signal Capture and Intelligent Processing, School of Electronic Engineering, Yili Normal University, 448 Jiefang Road, Yining 835000, China; 20230050200@ylnu.edu.cn (Y.P.); 20220050567@ylnu.edu.cn (Q.L.); 14012@ylnu.edu.cn (Y.F.); 20230050199@ylnu.edu.cn (H.C.); 20240050197@ylnu.edu.cn (D.Z.); 20220050568@ylnu.edu.cn (H.L.); 2 Key Laboratory of Intelligent Optical Sensing and Manipulation, College of Engineering & Applied Sciences, Ministry of Education, Nanjing University, 163 Xianlin Ave, Nanjing 210023, China; weijiang@nju.edu.cn

**Keywords:** signal processing, neural network, vibration signal, wavefront sensor

## Abstract

In geophysical exploration, laser remote sensing detection of seismic waves based on wavefront sensors can be used for geological detection and geophysical exploration. However, due to the high sensitivity of the wavefront sensor, it is easy to be affected by the environmental light and vibration, resulting in random noise, which is difficult to predict, thus significantly reducing the quality of the vibration signal and the detection accuracy. In this paper, a large amount of data is collected through a single-point vibration detection experiment, and the relationship between amplitude and spot centroid offset is analyzed and calculated. The real noisy vibration signal is denoised and signal enhanced by using a BiLSTM denoising convolutional self-encoder (BiL-DCAE). The irregular and unpredictable noise generated by various complex noise mixing is successfully suppressed, and its impact on the vibration signal is reduced. The signal-to-noise ratio of the signal is increased by 13.90 dB on average, and the noise power is reduced by 95.93%, which greatly improves the detection accuracy.

## 1. Introduction

Seismic wave laser remote sensing and detection technology has developed rapidly recently, providing new means for geological structure identification and resource exploration by virtue of the advantages of non-contact, high resolution, and long range [1,2,3]. Among many optical measurement techniques, wavefront sensors have received widespread attention due to their simple structure and flexible deployment [4,5]. Compared with traditional interferometers, wavefront sensors do not need to set up complicated reference arms and measurement arms, and the system is more compact, which is suitable for rapid deployment in complex environments in the field.

Wavefront sensors can capture ground vibration signals with high sensitivity by measuring small changes in the phase of the laser wavefront, with sub-millimeter displacement detection capability [6]. Meanwhile, its array structure supports multi-point simultaneous detection, which improves the efficiency of spatial information acquisition while maintaining high resolution. It is shown that the system based on the Shack–Hartmann wavefront sensor can accurately identify the displacement of point or medium-scale targets [7].

Compared with the traditional seismic wave remote sensing methods, the wavefront sensor shows obvious advantages in terms of detection accuracy, system volume, and field adaptability [8]. A laser remote sensing system for seismic waves based on wavefront sensors has been successfully constructed, which realizes long-range and high-precision measurements of weak vibration signals on the ground and demonstrates its enormous potential in resource exploration and deep structure analysis under complex geological conditions [9,10].

However, the use of a wavefront sensor for detection is often interfered with by many external factors. The external interference is the noise caused by environmental vibration, sunlight, and environmental wind. This kind of noise has substantial interference on the collected signal and will significantly reduce the signal-to-noise ratio of the signal [10]. At the same time, the wavefront sensor also has interference in the acquisition process, such as CMOS readout noise, photon shot noise, and sampling noise [11,12]. Internal noise usually has less impact. The above noises are usually random and mixed with each other, resulting in complex and unpredictable noises, which will significantly reduce the stability of the vibration signal. All the above signs show that removing the mixed noise in the vibration signal is particularly critical to the improvement of the remote sensing detection system.

The field seismic exploration data in complex acquisition environments usually has the characteristic of a low signal-to-noise ratio, and the nature of seismic exploration random noise in different survey areas will be affected by surface conditions and acquisition environments, showing a variety of complex noise characteristics, such as non-stationary and non-Gaussian, which makes the traditional filtering method difficult to adapt to complex seismic exploration data processing.

Traditional signal denoising methods, such as low-pass filters and wavelet transforms, are effective in some cases, but their effect is often unsatisfactory when dealing with complex non-stationary signals and strong interference noise [13]. This is because such methods usually rely on prior knowledge and experience to analyze the characteristics of noise and then remove it, which is difficult to apply to a variety of complex actual scenes.

With the rapid development of deep learning, neural networks have shown great potential in the field of signal processing. It can automatically learn features and recognize patterns and has been widely used in noise reduction tasks in voice, image, and other fields [14,15]. For example, many studies have shown that neural networks can effectively extract signal features from complex backgrounds, improve signal quality, and show superior noise reduction ability in processing vibration signals [16].

Firstly, in this paper, we obtained a large number of amplitude and spot centroid offset data through a single-point vibration experiment, fully verified the positive proportional relationship between spot centroid offset and amplitude, and calculated the positive correlation proportional coefficient.

Secondly, we design and apply the BiL-DCAE (Bidirectional LSTM-based Denoising Convolutional Autoencoder), a hybrid neural network architecture for vibration signal denoising, within the laser remote sensing detection system based on a wavefront sensor. Through a large number of collections and analyses of the noisy vibration information and clean vibration information detected by the system, the purpose is to explore the characteristics of the vibration signal so as to effectively reduce the noise of the noisy signal, remove the inevitable noise interference in the collection, and retain the vibration information of interest so as to restore the characteristics of the real vibration signal.

The experimental results show that the BiL-DCAE neural network architecture can remove most of the noise in the vibration signal, retain the characteristics of the vibration signal, and improve the indicators of the signal after noise reduction. The success of the experiment can enable the system to collect vibration signals without setting a shield, which greatly reduces the complexity and limitations of the system setting and provides a favorable means for the next step of outdoor operation.

## 2. Laser Remote Sensing System for Seismic Wave Detection

### 2.1. Working Principle of Wavefront Sensor

Seismic waves can be divided into P-waves, S-waves, and surface waves according to the mode of propagation. Among them, shear waves can only propagate in solids and often cause horizontal ground shaking, which is difficult to observe. Surface waves are mostly interference waves; because the P-wave is an advancing wave, it is the first wave to reach the surface, which can be clearly observed, and it is also the main detection object of the geophone.

**Figure 1:**
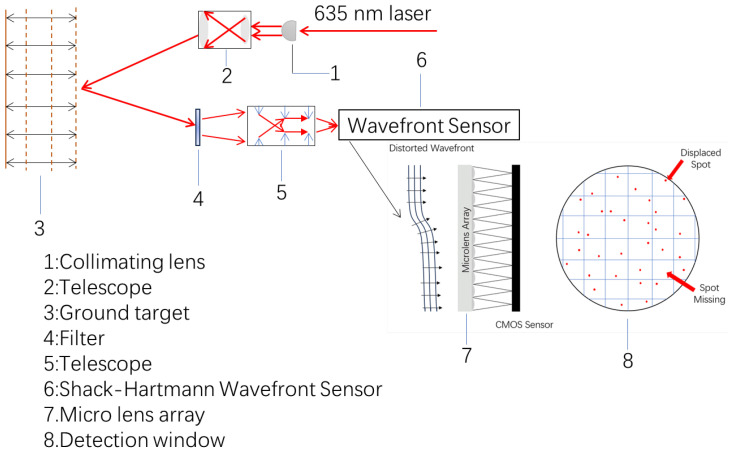
Vibration signal point scanning detection system.

In this paper, a point-scanning remote sensing system for vibration signal detection is designed, as shown in Figure 1, which consists of a transmitting end and a receiving end. The transmitting end consists of a laser, a collimator (1), and a telescope (2). The laser emits a continuous beam with a wavelength of 635 nm and constant power, which is collimated by a pigtail-type collimator equipped with a GRIN (Gradient-Index) lens, a lens with a radially varying refractive index that enables efficient beam collimation, to irradiate the target area at a stable angle and power (3). The receiving end consists of a telescope (5), a filter (4), and a wavefront sensor (6). The telescope receives light reflected and scattered from the target, and the filter removes ambient stray light to improve signal efficiency. The light then enters the wavefront sensor, which detects wavefront aberrations with high accuracy. The sensor contains an array of microlenses (7), each of which operates independently, and the array splits the incident light into multiple sub-apertures and focuses it onto individual spot detection windows (8).

### 2.2. Centroid Offset–Amplitude Relationship

**Figure 2:**
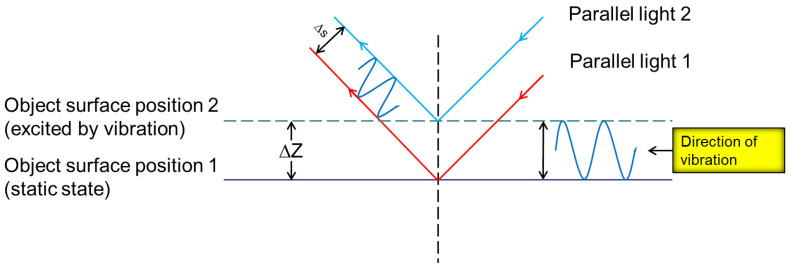
Relationship between surface amplitude and reflected laser.

The longitudinal wave will cause the ground to vibrate up and down, which can be regarded as the change of ground amplitude. In this paper, the vibroseis is used to excite the ground at different frequencies to change the amplitude of the ground, and then the receiving end of the wavefront sensor is placed perpendicular to the ground to simulate the influence of the longitudinal wave in the seismic wave on the laser wavefront. When the laser is reflected back from the ground, its wavefront will change and contain the vibration information of the ground. The measurement spot formed by the microlens array on the wavefront sensor will also change because the amount of light filling its aperture changes. The seismic wave information generated by ground excitation can be obtained by measuring the change of laser wavefront. When the target object is affected by different intensities of vibration, the change of the measurement spot is also different. The principle of measuring object vibration information using a wavefront sensor is shown in Figure 2.

The system uses the offset to obtain the vibration information and uses the laser remote sensing system to detect the structural micro vibration in the target area. It is necessary to understand the relationship between the phase information of the target vibration and the offset of the received spot in the CMOS sensor pixel.

**Figure 3:**
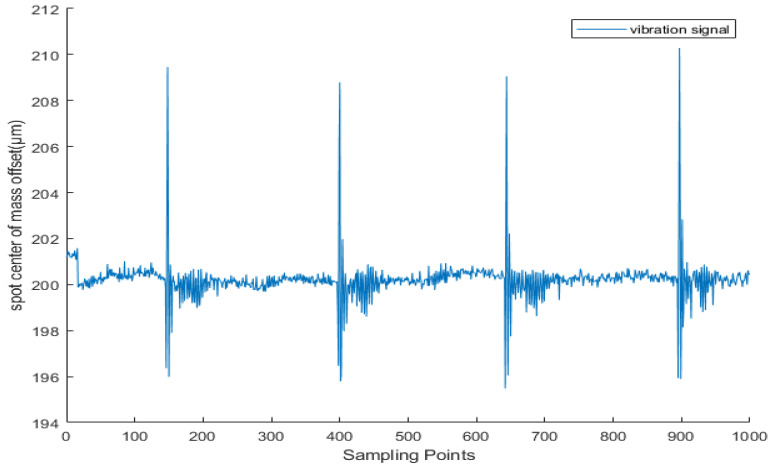
The offset of the centroid of the light spot captured during vibration occurrence.

It can be seen from Figure 2 that when the target area is externally excited, the position of the ground surface will change, the wavefront phase of the laser will also change, and the corresponding measurement spot position on the wavefront sensor will change. The vibration waveform is shown in Figure 3. The wavefront sensor spot centroid offset Δs carries vibration information Δz. It can be seen from the literature that the displacement change Δs of the measured spot is only related to the ground amplitude Δz. The greater the ground amplitude, the greater the displacement of the spot. The two are in positive proportion as shown below.

(1) Δz=μΔs

### 2.3. Noise Source Analysis

The Shack–Hartmann wavefront sensor is an instrument commonly used for optical wavefront measurement. It evaluates the wavefront distortion of the optical system by measuring the deflection of the beam passing through multiple small holes. It is widely used in astronomical observation, laser beam control, optical testing, and other fields. However, in the process of use, the signal collected by the sensor may be subject to a variety of interference. The following are the common types of interference and their causes.

Ambient light interference: as the Shack–Hartmann sensor is a very sensitive optical instrument, ambient light (such as sunlight, artificial light sources, etc.) may be mixed with the original signal, thus affecting the measurement accuracy. Ambient light will introduce additional noise to the optical signal collected by the sensor, interfering with its accurate measurement of wavefront distortion.

Noise caused by environmental vibration: In the laboratory environment, external mechanical vibration, sound, and ground vibration caused by crowd activities may cause slight displacement of the vibration source and sensor receiving end. These micro vibrations will make the centroid of the spot received by the wavefront sensor deviate, resulting in the deviation between the measured results and the actual values. Especially in the high-precision measurement scene, this kind of noise has a more significant impact on the wavefront measurement and then affects the stability and accuracy of the sensor.

**Figure 4:**
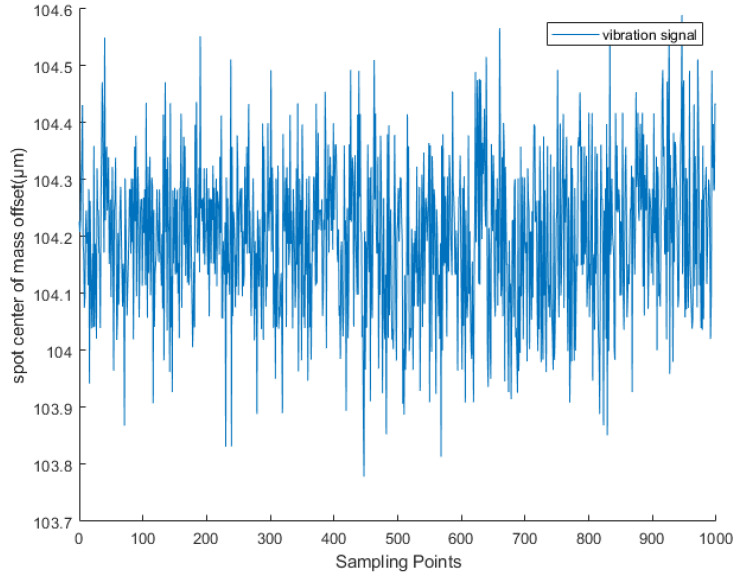
Noise waveform in an unobstructed exposure environment.

The above two types of interference often occur at the same time and overlap with each other, forming complex and unpredictable environmental noise as shown in Figure 4. Such noise will significantly reduce the signal quality. In order to solve this problem, the solutions we adopt include using a shield to isolate the interference of background light and placing the sensor in a shielded environment so as to eliminate the influence of ambient light on the measurement.

## 3. Theoretical Framework

### 3.1. Wavefront Sensor Principles and Formulas

**Figure 5:**
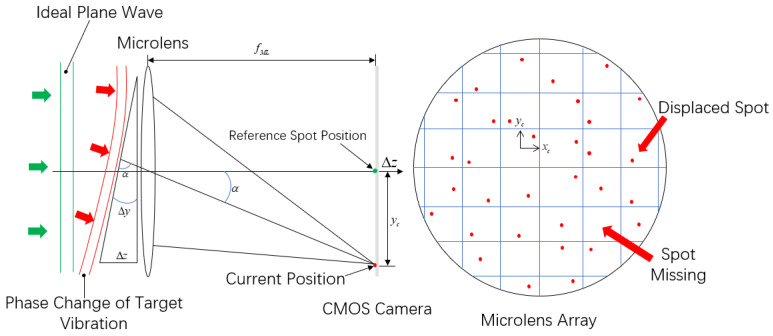
Wavefront distortion of wavefront sensor micro lens array. When parallel wavefronts enter, the centroid points of all light spots are at the center of the microlens array. When distorted wavefronts enter, the centroid points of the light spots will shift from the center or even disappear.

The wavefront sensor employed in this study consists primarily of a microlens array, relay optics, and a CMOS imaging sensor. When a coherent beam impinges upon the system, the microlens array spatially segments the wavefront into discrete sub-apertures. Each microlens focuses its portion of the beam onto the CMOS plane, forming an array of focal spots. Under ideal planar wavefront conditions, these spots are symmetrically aligned. However, any wavefront aberration leads to deviations in spot positions, which are indicative of local wavefront slopes. These slopes can be quantitatively retrieved through centroid detection algorithms as shown in Figure 5.

When the target surface experiences vibration, the incident beam’s reflection or scattering angles are altered, inducing spatial phase distortions in the returned wavefront. Such distortions manifest as displacements of the focal spot array on the sensor, enabling indirect capture of the vibrational phase information.

In the experiment on laser-based detection of seismic longitudinal wave vibrations, a Shack–Hartmann wavefront sensor (SHWFS)—an optical device that measures wavefront distortions by analyzing the displacement of focal spots formed by a microlens array—is employed. The SHWFS used in this study features high-speed mode and high frame rate sampling, enabling it to effectively respond to rapid and subtle variations in the laser signal. It can directly measure the laser wavefront without coaxial processing of the optical platform, greatly facilitating the sampling work. The wavefront sensor consists of two parts: a microlens array and a CMOS sensor. The measured wavefront is sampled by the microlens array and then displayed by the sensor. When a laser beam passes through a microlens array, each microlens collects the amount of light filling its aperture and forms a single focal point on the CMOS sensor at the focal plane of the microlens array. If the wavefront is flat, the focal point is located at the center of the optical axis of each lens, which is called the reference spot. If the wavefront undergoes distortion, the focal point will deviate from the reference spot position on the CMOS sensor, which is called the measurement spot. The imaging principle of a single microlens is shown in Figure 5. By comparing the positional changes between the measured spot and the reference spot, the phase change of the laser wavefront can be analyzed and calculated. A detailed mathematical formulation of the SHWFS principle is available in the work of Rodier [17], where the microlens array is modeled as a two-dimensional phase grating, linking wavefront distortions to spot displacements on the sensor. In this paper, we have supplemented the derivation and related formulas in Appendix A.

### 3.2. Model Components

Denoising Convolutional Autoencoders (DCAEs), which combine convolutional encoding with noise-robust training, have been widely used for signal denoising due to their effective local feature extraction [18]. However, their ability to capture long-term temporal dependencies in sequential data is limited. To address this, recurrent neural networks (RNNs), especially Long Short-Term Memory (LSTM) and Bidirectional LSTM (BiLSTM) variants, have been integrated into autoencoder frameworks, enhancing temporal modeling by processing sequences in forward and backward directions [19,20,21].

**Figure 6:**
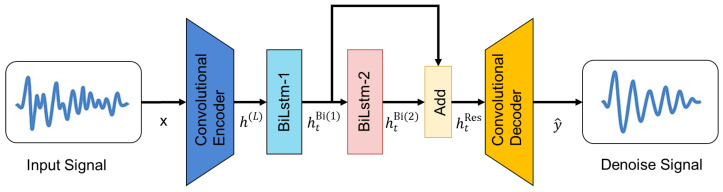
Overall architecture diagram of BiL-DCAE.

Based on this motivation, we propose a novel Bidirectional LSTM-enhanced Denoising Convolutional Autoencoder (BiL-DCAE) for seismic signal denoising. The overall architecture is shown in Figure 6. It consists of three main components: a convolutional encoder, a residual BiLSTM module, and a convolutional decoder.

Convolutional Encoder: The encoder extracts hierarchical local features from the noisy input signal. For an input sequence $x\in R^{T}$, the convolutional encoder applies a series of 1D convolutional operations:

(2) $$h^{\left(l\right)}=\sigma \left(W^{\left(l\right)}*h^{\left(l-1\right)}+b^{\left(l\right)}\right),\;l=1,\ldots ,L$$

where $h^{\left(0\right)}=x$, $W^{\left(l\right)}$, and $b^{\left(l\right)}$ denote the kernel and bias of the *l*-th layer, *l* denotes convolution, and σ(·) is the activation function (ReLU in our case).

Residual BiLSTM Module: To capture temporal dependencies in both the forward and backward directions, we insert a BiLSTM layer after the encoder. The BiLSTM processes the encoded feature sequence $h^{\left(L\right)}$ as

(3) $${\vec{h}}_{t}=\mathrm{LSTM}\left(h_{t}^{\left(L\right)},{\vec{h}}_{t-1}\right),\;{\overset{\leftarrow }{h}}_{t}=\mathrm{LSTM}\left(h_{t}^{\left(L\right)},{\overset{\leftarrow }{h}}_{t+1}\right)h_{t}^{\mathrm{Bi}}=\left[{\vec{h}}_{t};{\overset{\leftarrow }{h}}_{t}\right]$$

Then, a second BiLSTM layer is applied to refine the temporal features:

(4) $$h_{t}^{\mathrm{Bi}(2)}=\mathrm{BiLSTM}\left(h_{t}^{\mathrm{Bi}(1)}\right)$$

To enhance feature propagation and stabilize gradient flow, we introduce a residual connection between the two BiLSTM layers:

(5) $$h_{t}^{\mathrm{Res}}=h_{t}^{\mathrm{Bi}(2)}+h_{t}^{\mathrm{Bi}(1)}$$

This residual connection preserves low-level temporal information while allowing the deeper BiLSTM to focus on modeling higher-level dependencies.

Convolutional Decoder: The decoder reconstructs the denoised signal from the BiLSTM-enhanced features using transposed convolutional layers:

(6) $$\hat{y}=\phi \left(U^{\left(m\right)}*h^{\mathrm{Bi}}+c^{\left(m\right)}\right),\;m=1,\ldots ,M$$

where $U^{\left(m\right)}$ and $c^{\left(m\right)}$ denote the decoder kernel and bias, and ϕ(·) is a linear activation at the output layer.

Loss Function and Training: The model is trained end-to-end by minimizing the mean square error (MSE) between the reconstructed signal $\hat{y}$ and the clean reference signal y:

(7) $$L_{\mathrm{MSE}}=\frac{1}{N}\sum_{i=1}^{N}{\left(y_{i}-\hat{y_{i}}\right)}^{2}$$

where y and $\hat{y}$ represent the *i*-th sample point of the clean and reconstructed signals, respectively, and *N* denotes the total number of sampling points in each signal segment.

Compared with conventional DCAE models, the proposed BiL-DCAE introduces a Bidirectional LSTM (BiLSTM) module that captures long-range dependencies in both the forward and backward directions, thereby enhancing the network’s ability to model complex temporal correlations in seismic signals. To further improve feature propagation and stabilize training, a residual pathway is incorporated between the stacked BiLSTM layers, enabling the network to learn richer temporal representations without performance degradation. This hybrid architecture integrates the strengths of convolutional feature extraction, residual-enhanced recurrent modeling, and end-to-end optimization, making it particularly suitable for processing and denoising ground vibration signals acquired by wavefront sensors in laser-based seismic remote sensing applications.

Key innovations and advantages: The proposed BiL-DCAE model innovatively integrates convolutional encoder–decoder layers with a residual bidirectional LSTM bottleneck, leveraging the complementary strengths of local feature extraction and bidirectional long-range temporal modeling. Compared to traditional Denoising Convolutional Autoencoders (DCAE), which rely solely on local convolutional features without modeling long-term dependencies, BiL-DCAE significantly overcomes these limitations. In contrast to pure RNN, GRU, standard autoencoder, or BiLSTM models, our method effectively extracts hierarchical local features via convolutional layers while capturing bidirectional temporal correlations, thereby enhancing modeling capability for complex signals. Compared with Transformer-based models, BiL-DCAE offers greater stability and training efficiency when processing small-scale vibration signals and avoids the sensitivity of Transformers to hyperparameters and data distribution. Furthermore, the introduction of residual connections between stacked BiLSTM layers alleviates gradient vanishing, improves feature propagation, and stabilizes training, enabling the network to learn richer temporal representations. This design collectively enhances denoising performance and robustness across varying noise conditions. The effectiveness and novelty of this architecture are further demonstrated through comprehensive performance comparisons in subsequent sections.

## 4. Single-Point Vibration Detection and Dataset Construction

### 4.1. μ Value Fitting in Single-Point Vibration Detection

**Figure 7:**
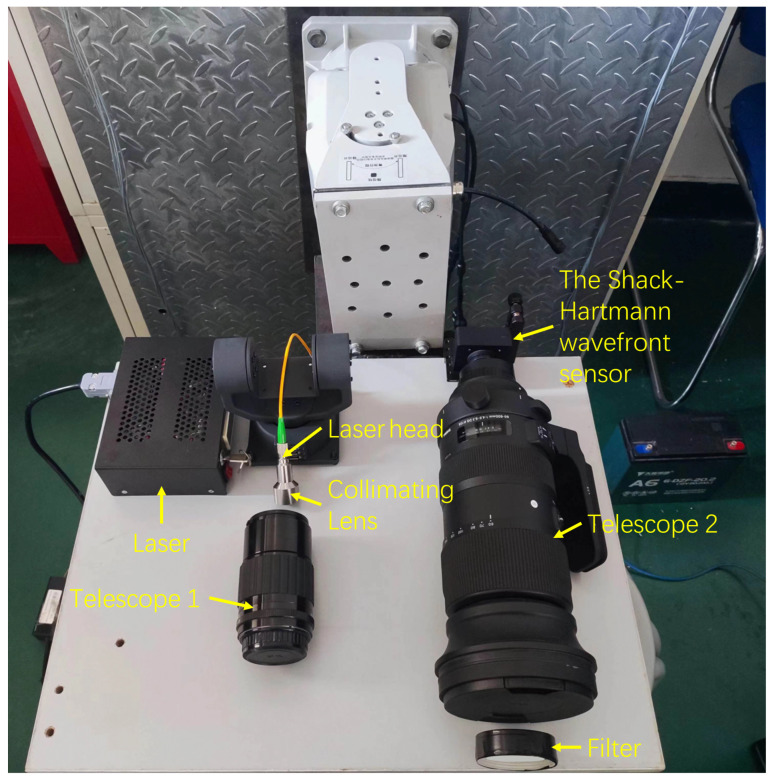
Physical image of the entire detection system.

In this experiment, a laser with an output power of 67 mW was used to simulate and detect longitudinal seismic vibrations over a distance of approximately 10 m. A photograph of the complete experimental setup is shown in Figure 7. To minimize external optical interference and ensure system stability, all devices were placed inside a shielding enclosure.

**Figure 8:**
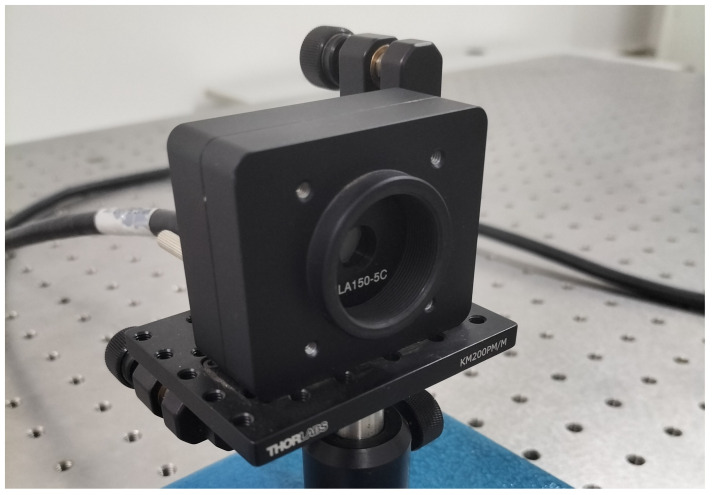
The WFS-20-5C Shack–Hartmann wavefront sensor.

The Shack–Hartmann wavefront sensor (model WFS-20-5C) manufactured by Thorlabs is used to detect vibration signals. This sensor offers a wavefront accuracy of λ/30rms@633nm and features a high sampling rate. With its built-in automatic shutter control, it is capable of handling a wide dynamic range of optical input power and maintains high sensitivity across different wavelengths. The physical diagram of the wavefront sensor is shown in Figure 8, and its detailed parameters are shown in Table 1. This sensor was utilized to detect laser wavefront distortions caused by surface vibrations on the target.

**Figure 9:**
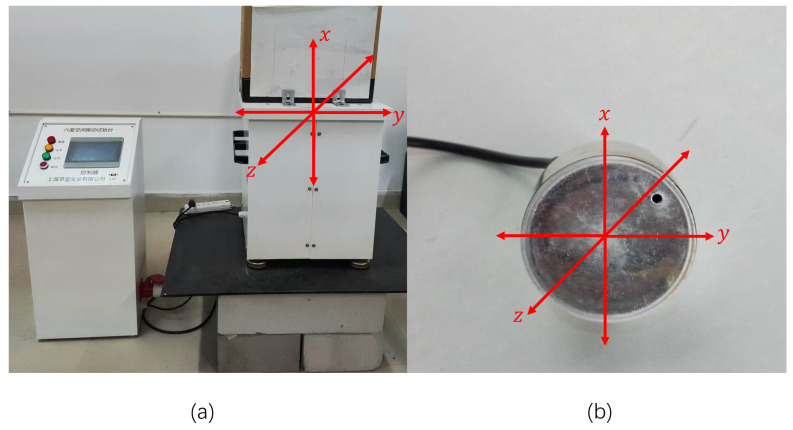
Controlled vibration table in the laboratory, serving as the excitation source of vibration signals: (**a**) controllable vibration table; (**b**) vibration motor. The x, y, and z directions marked in the figure indicate the vibration directions generated by the vibration device.

For the point-based vibration detection test, an electronically controlled vibration platform was used as the excitation source. By adjusting the platform’s frequency and amplitude, we were able to simulate typical longitudinal wave characteristics as found in natural seismic events. The physical appearance of the vibration source is shown in Figure 9. During the experiment, the laser beam was directed onto the vibrating surface, and the reflected beam was captured by the wavefront sensor to measure the resulting wavefront changes.

**Figure 10:**
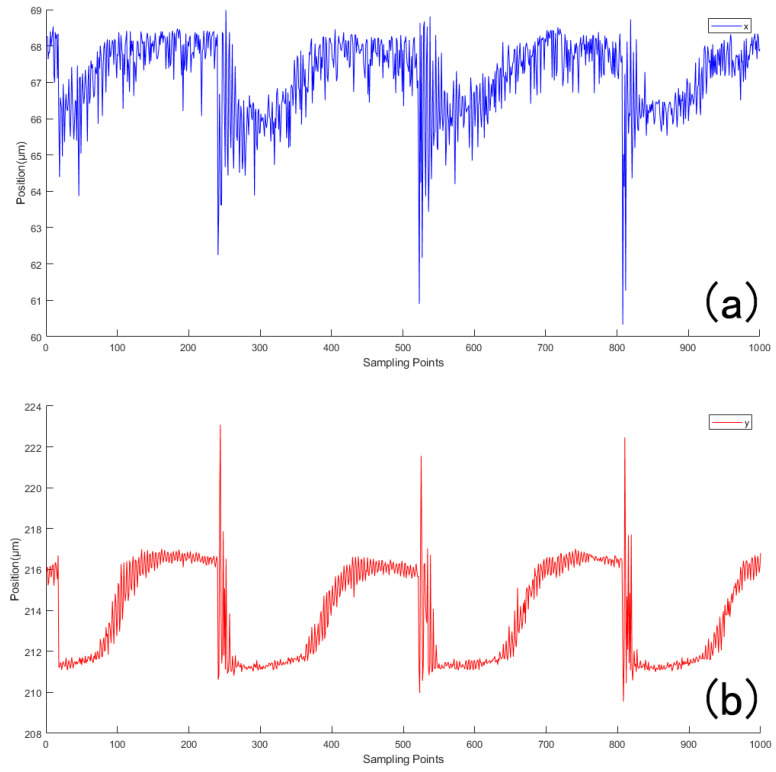
When the vibration source is excited, the displacement of the center of mass of the light spot changes. (**a**) Waveform of the microlens center-of-mass displacement x in the horizontal direction during vibration; (**b**) waveform of the microlens center-of-mass displacement y in the vertical direction during vibration.

**Figure A2:**
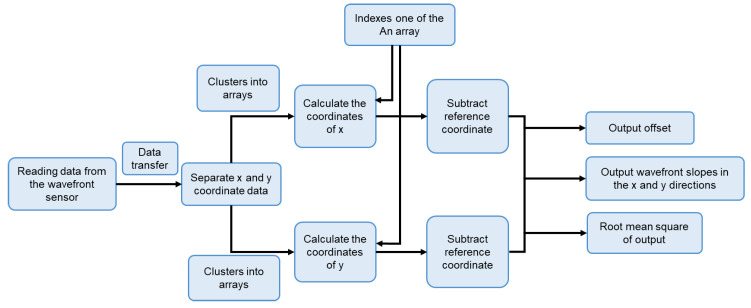
Calculation process.

To enable real-time observation of the spot centroid displacements on the sensor’s microlens focal plane, we used the Wavefront Sensor software (version 18183-D03) to continuously monitor and record the centroid shifts. The software workflow is shown in Figure A2 of Appendix B. As shown in Figure 10, the vibration source induced visible displacements in both the horizontal and vertical directions, with the displacement along the y-axis being significantly larger.

**Figure 11:**
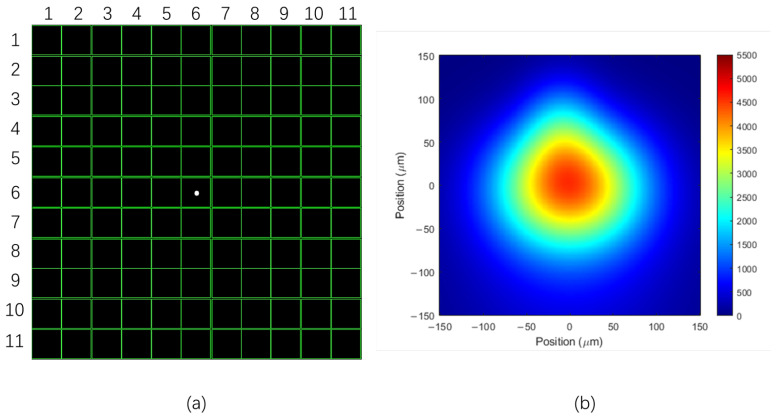
Single microlenses in a microlens array receive light spots. (**a**) The spot signal of the 6th × 6th microlens in high-speed sampling mode; (**b**) the spot signal of the 6th × 6th microlens in beam view mode.

For experiments on the reception point of a single microlens, the laser echo signal is focused through the telescope on one of the microlenses in the microlens array. The high-speed sampling mode in wavefront sensor software is a feature that enables the real-time acquisition and analysis of wavefront data. This mode captures wavefront data at a high sampling rate, allowing the system to monitor rapidly changing wavefronts in real time, particularly suitable for dynamic scenes or fast-moving objects. The beam view mode of a wavefront sensor is a display mode used to observe and analyze the beam distribution on the sensor. In this mode, the output signal of the wavefront sensor will be displayed in the form of a beam, usually in the form of an image or visual light spot. This mode allows for intuitive observation of the characteristics of the beam, such as the shape, size, and position of the light spot. In the beam view mode, various characteristics of the beam can determine the alignment of the optical system, especially whether there are issues such as focal length offset, beam extension, and skewness. In the high-speed sampling mode of the wavefront sensor software, a clear spot centroid was observed at the center of the microlens array as shown in Figure 11a, and Figure 11b in the beam view mode of the wavefront sensor.

The specific operation is as follows: the laser forms a light spot on the controllable vibration table, and the echo signal is focused by the telescope to the 6th × 6th microlens of the wavefront sensor. LabView software was used to obtain the offset of the centroid of the 6th × 6th micro lens spot.

We captured the proportional relationship between the offset of the center of mass of the light spot and the amplitude through the single-point vibration test above. The larger the offset of the center of mass of the light spot, the larger the amplitude.

**Figure 12:**
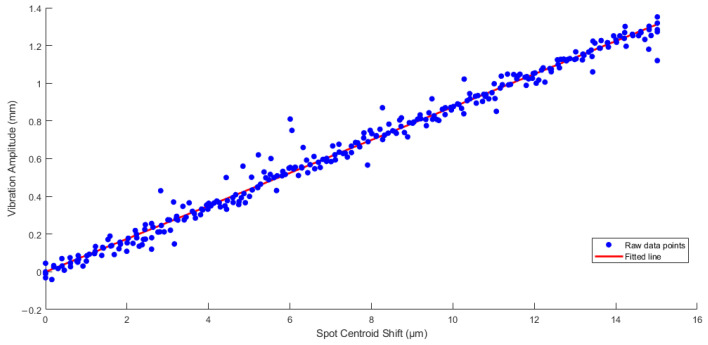
The fitting line graph of μ was obtained by fitting 287 vibration signal data and corresponding amplitudes collected by the vibration meter, and the fitted μ was calculated to be 0.0874.

Then, by fitting the collected vibration signals to their corresponding spot centroid offsets, we obtained the relationship μ between amplitude and spot centroid offsets as shown in Figure 12.

### 4.2. Real Vibration Signal Dataset

**Figure 13:**
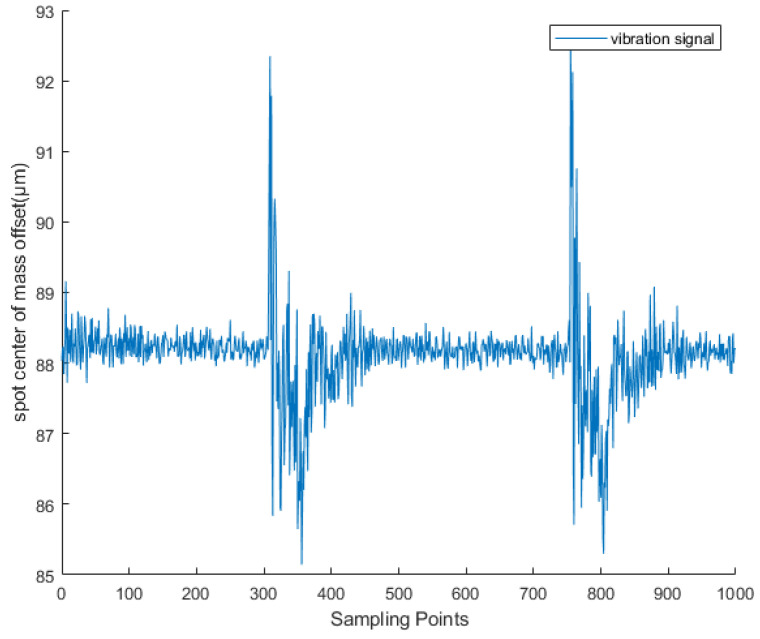
Clean vibration signals captured by wavefront sensors in a laboratory environment using a shielding cover when excited by a vibration source.

**Figure 14:**
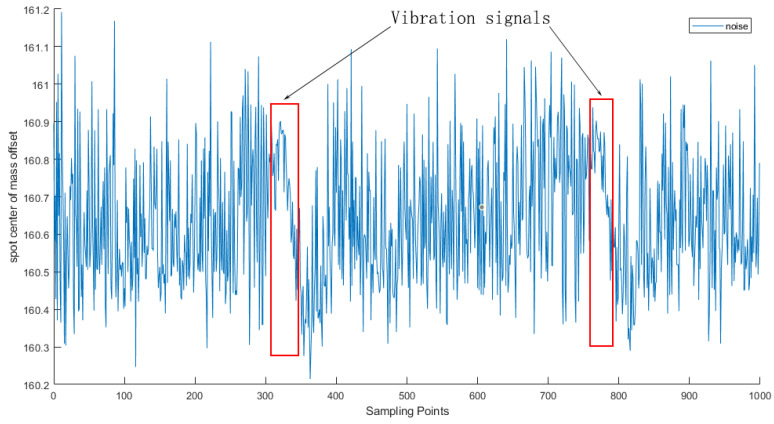
When the vibration source is excited, the waveform of the centroid offset of the light spot captured by the acquisition system equipment (wavefront sensor) exposed to the laboratory environment reflects the vibration signal with noise, and the marked part is the vibration signal. It can be seen that the signal is almost submerged in complex and diverse noise.

**Figure 15:**
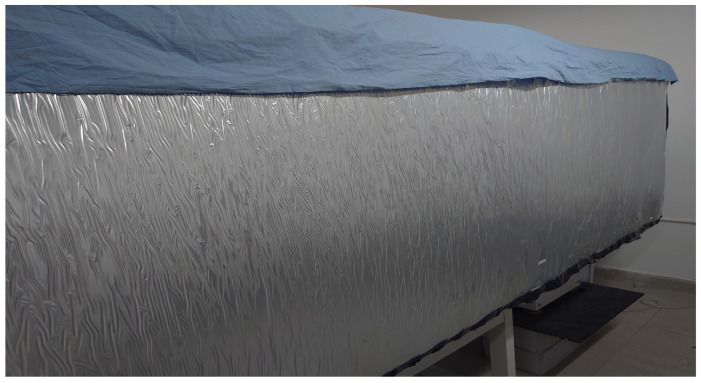
Physical picture of the shielding cover.

Based on extensive data collected via LabVIEW software (version LabView2020.0 32-bit) during single-point vibration detection experiments, the dataset used for neural network training is divided into two categories depending on the use of a shielding cover. The clean signals were captured at night under a laboratory shielding mask, where ambient light and environmental vibrations were minimal, providing near-ideal acquisition conditions (Figure 13). In contrast, the noisy signals were recorded under the same experimental setup but without any shielding, allowing natural environmental interference to affect the measurements as illustrated in Figure 14. Both types of signals were acquired using a wavefront sensor (sampling frequency: 28.5 Hz), which was determined through extensive testing to provide the most stable and reliable data acquisition for our vibration detection system. Each data file contains 1000 sampling points. The shielding device used is shown in Figure 15.

All datasets used in this study are composed entirely of real-world vibration signals obtained from the above-described experimental system. No synthetic signals were used. To ensure the diversity and representativeness of the dataset, signals were collected at a series of controlled vibration amplitudes (i.e., 0.06, 0.12, 0.18, 0.25, 0.31, 0.37, 0.43, 0.50, 0.56, 0.62, 0.75, 0.81, 0.87, 0.93, 1.00, 1.06, 1.12, and 1.18 mm) and discrete frequencies (0.1 Hz, 0.5 Hz, and 1 Hz). A total of 5150 samples were randomly selected as the test set, with the remainder used for training. This setup ensures the dataset captures both ideal and noisy laboratory conditions, supporting robust model training and evaluation. The complete dataset composition is summarized in Table 2.

Specifically, during dataset construction, each collected signal *x* is standardized using z-score normalization, where the standardized value $x^{\prime }$ is computed as

(8) $$x^{\prime }=\frac{x-\mu }{\sigma },$$

with μ and σ denoting the mean and standard deviation of the signal, respectively. This process not only ensures zero mean and unit variance, improving consistency and numerical stability for subsequent model training, but also effectively mitigates baseline drift. Such drift may arise during long-term sensor operation due to thermal fluctuations, electronic bias changes, or minor mechanical deformations, causing gradual shifts of the signal baseline away from zero. By applying z-score normalization at this stage, these offsets are removed, allowing the model to better focus on the true vibration patterns.

All signals were preprocessed using z-score normalization to eliminate baseline drift and ensure consistency across the dataset. This step does not introduce synthetic information but rather enhances the reliability and reproducibility of the dataset for model training.

The dataset is not publicly available at this time, as it will serve as the basis for ongoing and future research. However, the acquisition process and sensor settings have been described in detail to ensure experimental reproducibility.

## 5. BiL-DCAE Denoising Experiment

The overall experimental workflow is structured into three sequential stages: dataset construction, model training, and model inference.

In the first stage, real-world vibration signals are acquired and then standardized using z-score normalization to eliminate baseline drift and ensure consistency in data distribution.

The dataset is first split into training and testing subsets, with 10% of the training data randomly selected as a validation set to monitor model performance during training.

Next, the model is trained using the prepared dataset, which is preprocessed through a series of transformation steps. The denoising model, initialized with a defined architecture, loss function, and hyperparameters, iteratively updates its parameters by minimizing reconstruction loss, while validation metrics are monitored to prevent overfitting. Upon training completion, the final model parameters are saved for later use.

Finally, the trained model is applied to unseen noisy vibration signals for inference. Input signals undergo the same preprocessing as during training to ensure consistency. The model then performs forward passes to generate denoised outputs, which are saved for further analysis or application.

All experiments were conducted on a server equipped with an RTX3090 GPU, significantly accelerating training time. The server configuration is detailed in Table 3. Python 3.8 and TensorFlow 2.13.0 were used for software development and model training.

**Figure 16:**
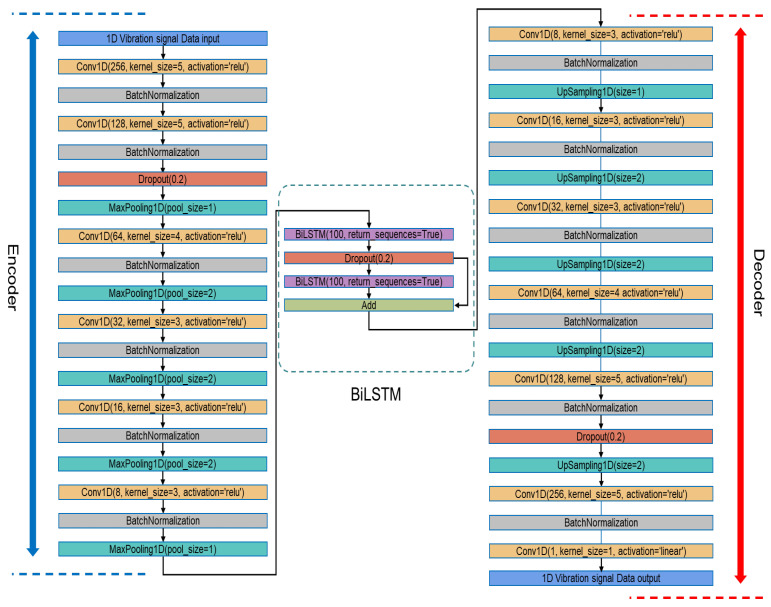
BiL-DCAE model network structure diagram.

The architecture of BiL-DCAE is shown in Figure 16. The encoder comprises six consecutive 1D convolutional layers with ReLU activation and mixed kernel sizes (5, 5, 4, 3, 3, 3), where the number of filters gradually decreases from 256 to 8. Each convolutional layer is followed by batch normalization, and MaxPooling1D layers with varying pooling sizes (1 or 2) are interleaved to perform temporal downsampling. Dropout layers are selectively applied to prevent overfitting.

After feature extraction, a stack of two bidirectional Long Short-Term Memory (BiLSTM) layers is used to capture bidirectional temporal dependencies. A residual skip connection is inserted between these two BiLSTM layers, allowing the output of the first BiLSTM to bypass the second and be added back to its output, improving gradient flow and enhancing the modeling of long-range dependencies.

The decoder mirrors the encoder with a symmetric structure of upsampling and convolutional layers. UpSampling1D layers with factors of 1 or 2 are alternated to gradually restore the temporal resolution, while the number of filters increases from 8 back to 256. Batch normalization is applied after each convolutional layer. Finally, a linearly activated 1D convolution layer reconstructs the output to a single-channel denoised signal.

To systematically optimize the network architecture and training hyperparameters, the Hyperopt framework was employed with a Tree-structured Parzen Estimator (TPE) algorithm for Bayesian optimization. This approach aligns with prior studies that emphasize the importance of automated architecture selection in neural network design, such as Broad Bayesian learning for nonparametric modeling [22], genetic algorithm-based network optimization [23], and CNN architecture optimization for structural response estimation [24]. The search space included the number of convolutional layers (3–5), convolution kernel sizes (3, 5, 7), BiLSTM units (64–256), dropout rates (0.2–0.5), and learning rates (1 × $10^{-4}$ to 1 × $10^{-2}$). Iterative search was conducted to minimize validation loss. The final configuration comprises six convolutional layers (kernel sizes 5/5/4/3/3/3), two BiLSTM layers with 100 units each, dropout rate 0.2–0.3, and a learning rate of 1 × $10^{-3}$.

The model was trained using the Adam optimizer with an initial learning rate of 0.001 and mean square error (MSE) as the loss function. Training spanned 500 epochs with a batch size of 256. ModelCheckpoint and ReduceLROnPlateau were used to preserve the best model and adaptively adjust the learning rate, respectively. TensorBoard was utilized for visualization of loss curves and weight dynamics.

**Figure 17:**
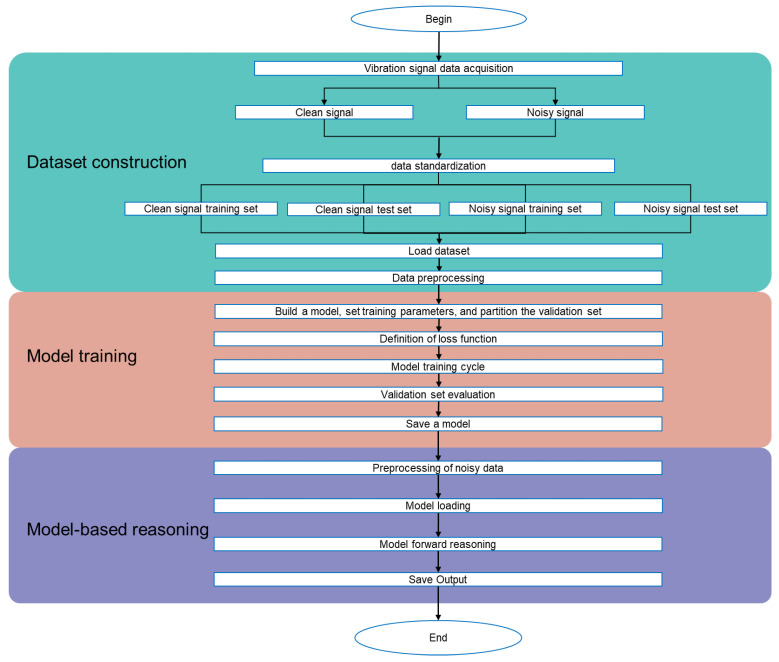
Experimental flowchart.

The experimental workflow is illustrated in Figure 17, and the optimal hyperparameter configuration obtained through Hyperopt is summarized in Table 4.

**Figure 18:**
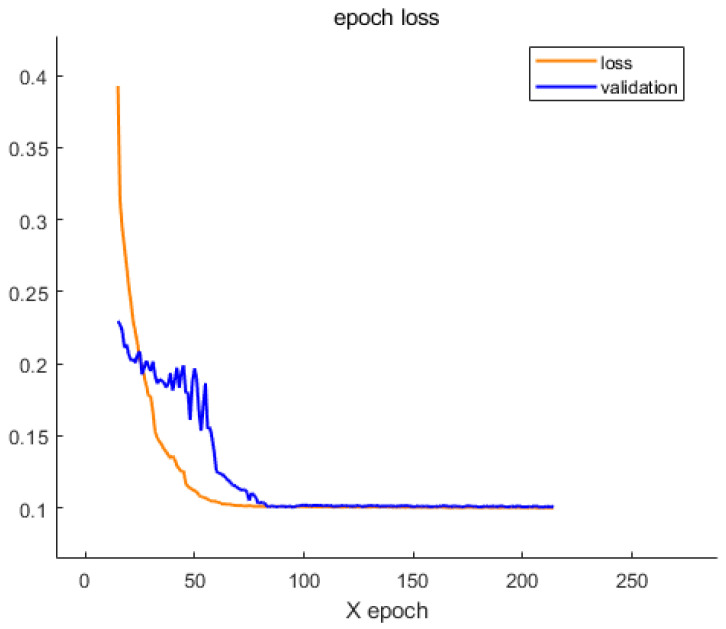
Training and validation loss curves over 200 epochs, demonstrating the convergence behavior of the proposed model.

To evaluate the training dynamics and convergence behavior of the proposed model, the loss values on both the training and validation sets were monitored over 200 epochs. As illustrated in Figure 18, the training loss consistently decreases during the initial epochs, indicating effective learning of the underlying signal features. However, after approximately 100 epochs, both training and validation losses begin to plateau, suggesting that the model has reached a stable fitting state and further training yields limited improvement.

## 6. Performance Evaluation

In this paper, a variety of evaluation indicators are used to comprehensively measure the signal before and after noise reduction. Specifically, the effective model saved after the training is used to reason the test set and calculate and evaluate the indicators of the noise reduction signal and the original clean signal obtained by the reasoning. At the same time, through the time–frequency analysis of the noise reduction signal and the original signal, the experimental results are fully displayed. The predicted value in the evaluation index refers to the signal after noise reduction (i.e., model output signal), while the true value is the original clean signal (noise-free signal).

### 6.1. Evaluation Metrics

**Figure 19:**
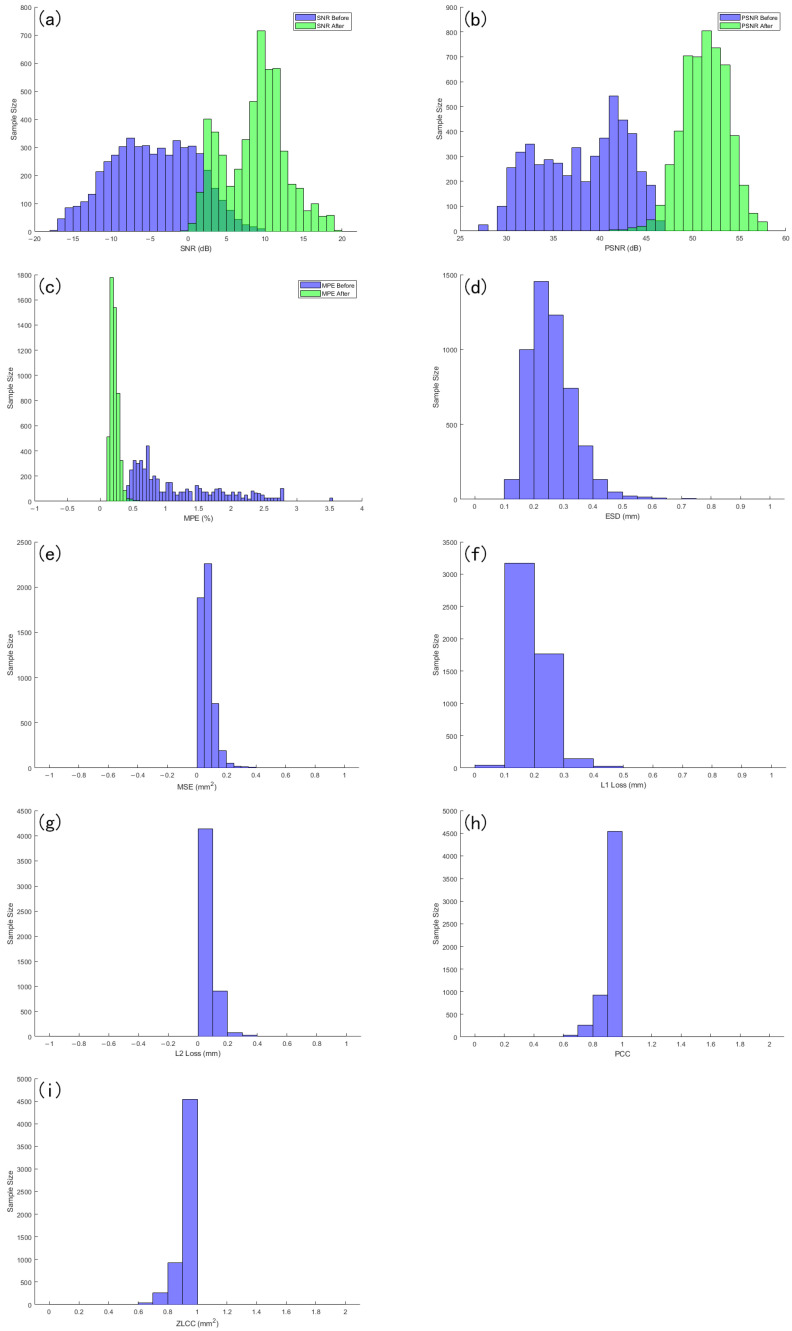
This figure shows the statistical distribution of the proposed denoising method on nine performance indicators, including (**a**) SNR, (**b**) PSNR, (**c**) MPE, (**d**) ESD, (**e**) MSE, (**f**) L1 Loss, (**g**) L2 Loss, (**h**) PCC, and (**i**) ZLCC. Each subgraph is represented in the form of a histogram, with the horizontal axis representing the numerical range of the corresponding indicator and the vertical axis representing the number of samples falling within that range. In the figure, (**a**–**c**) simultaneously present the distribution comparison of indicators before and after denoising, and (**d**) shows the statistical characteristics of each indicator after denoising treatment.

SNR (signal-to-noise ratio) [25]: In this manuscript, the SNR is defined here for the first time. SNR measures the ratio between signal power and noise power. It is usually used to represent the strength of a signal in a noisy environment. The higher the SNR value, the stronger the useful information in the signal is compared with the noise, which indicates that the quality of the signal is better:

(9) $$\mathrm{SNR}=10\cdot \mathrm{lo}g_{10}\left(\frac{P_{\mathrm{signal}}}{P_{\mathrm{noise}}}\right)$$

where $P_{\mathrm{signal}}$ is the power of the signal and $P_{\mathrm{noise}}$ is the power of the noise. In practical applications, the power of signal and noise is usually estimated by its mean square value. To assess the overall denoising performance of the proposed model across the entire dataset, a histogram-based comparison was conducted based on the signal-to-noise ratio (SNR). As illustrated in Figure 19a, the x-axis represents the SNR values, while the y-axis indicates the number of samples corresponding to each SNR range. The results reveal a significant rightward shift in the distribution after denoising, demonstrating that a majority of the signals achieved higher SNR values compared to their original noisy counterparts. This confirms that the model effectively enhances the signal quality and reduces noise across a wide range of input conditions.

PSNR (peak signal to noise ratio) [26]: PSNR is an index to measure the peak signal-to-noise ratio between the denoised signal and the clean signal. The basic idea is to reflect the difference between the denoised signal and the original clean signal through the mean square error (MSE) and convert it into a signal-to-noise ratio. The higher the PSNR value, the better the reconstruction quality of the image or signal:

(10) $$\mathrm{PSNR}=10\cdot \log_{10}\left(\frac{MAX_{I}^{2}}{MSE}\right)$$

where $MAX_{I}$ is the maximum possible value of the signal and MSE is the mean square error.

Figure 19b shows the sample size distribution of the signal before and after denoising in different PSNR intervals. The horizontal axis represents PSNR values, and the vertical axis represents the corresponding number of samples in each interval. As can be seen from the figure, the PSNR before denoising is mostly concentrated in the lower numerical range, with a relatively scattered distribution; the PSNR after denoising showed a significant right shift overall, and the number of samples increased significantly in the higher PSNR range, indicating that denoising effectively improved signal quality. In most samples, the denoising operation resulted in significant PSNR gain, demonstrating the advantage of the proposed method in signal fidelity.

MPE (mean percentage error) [27]: MPE is used to measure the relative error between the reconstructed signal and the clean signal. It calculates the ratio of the error of each point to the real signal and averages all data points. MPE can provide the relative size of error, which is helpful to evaluate the performance of the noise reduction model:

(11) $$\mathrm{MPE}=\frac{1}{N}\sum_{i=1}^{N}\frac{|x_{\mathrm{true}}\left[i\right]-x_{\mathrm{pred}}\left[i\right]|}{|x_{\mathrm{true}}\left[i\right]|}\times 100\%$$

where $x_{\mathrm{true}}\left[i\right]$ and $x_{\mathrm{pred}}\left[i\right]$ are the real signal and reconstructed signals of the ith point, respectively.

Figure 19c shows the sample size distribution of the signal before and after denoising in different MPE intervals. The horizontal axis represents the MPE value, and the vertical axis represents the number of samples corresponding to each interval. It can be seen that the MPE distribution before noise reduction is generally biased towards larger numerical ranges, indicating that the original signal has a large error; after denoising, the MPE distribution clearly shifts towards the low value range, with a higher degree of concentration and a significant reduction in high error samples. This change indicates that the noise reduction method used effectively reduces the relative error of the signal and improves the accuracy and consistency of signal reconstruction.

ESD (error standard deviation) [28]: ESD measures the standard deviation of signal reconstruction error, which reflects the fluctuation of error in the whole dataset. The smaller the ESD value, the smaller the fluctuation of reconstruction error and the better the denoising effect:

(12) $$\mathrm{ESD}=\sqrt{\frac{1}{N}\sum_{i=1}^{N}{\left(x_{\mathrm{true}}\left[i\right]-x_{\mathrm{pred}}\left[i\right]\right)}^{2}}$$

ESD is mainly used to evaluate the stability and consistency of the model in the process of signal reconstruction.

Figure 19d shows the ESD distribution of the denoised signal, with the horizontal axis representing the numerical value of ESD after denoising and the vertical axis representing the number of samples in the corresponding interval. Overall, the ESD values of most samples are distributed in the lower range, and the histogram shows a concentrated distribution feature, indicating that the envelope spectrum of the denoised signal is closer to the reference signal, and the degree of spectral distortion is smaller. This indicates that the proposed denoising method has good performance in preserving the spectral features of the signal.

MSE (mean square error) [29]: MSE is a commonly used error measurement method, which is used to measure the difference between the real signal and the reconstructed signal. It can effectively quantify the accuracy of signal reconstruction by calculating the square of the error of each sample point and obtaining the mean value. The smaller the MSE, the smaller the difference between the reconstructed signal and the original signal:

(13) $$\mathrm{MSE}=\frac{1}{N}\sum_{i=1}^{N}{\left(x_{\mathrm{true}}\left[i\right]-x_{\mathrm{pred}}\left[i\right]\right)}^{2}$$

where $x_{\mathrm{true}}$ and $x_{\mathrm{pred}}$ are the signal and the reconstructed signal, respectively, and *N* is the total number of samples of the signal.

Figure 19e shows the distribution of mean square error (MSE) of the denoised signal in different numerical ranges. The horizontal axis represents the interval in which the MSE value is located, and the vertical axis represents the number of samples within the corresponding interval. From the graph, it can be seen that the MSE values of most samples are concentrated in the lower range, and the histogram shows a clear leftward distribution trend, indicating that the error between the denoised signal and the reference signal is relatively small.

L1 loss (mean absolute error) [30] calculates the average absolute value of the signal reconstruction error, which is mainly used to measure the accuracy of the model. Unlike MSE, L1 loss is not sensitive to outliers. The smaller the L1 loss value, the smaller the error between the reconstructed signal and the original signal:

(14) $$L1\mathrm{Loss}=\frac{1}{N}\sum_{i=1}^{N}\left|x_{\mathrm{truc}}\left[i\right]-x_{\mathrm{pred}}\left[i\right]\right|$$

L1 loss is mainly used for regression tasks and signal denoising, especially in the presence of large noise.

From Figure 19f, it can be intuitively seen that the error values are mainly distributed in the range close to zero, and the absolute error of most samples remains at a low level, with only a small amount distributed in the high-value area. This distribution pattern indicates that after denoising treatment, the model can accurately restore the signal in most cases, and the error fluctuates little between samples. In other words, the low value set of L1 Loss not only reflects the improvement of denoising accuracy but also demonstrates the good robustness of the method under different sample conditions.

L2 loss (mean square error) [31]: L2 loss is a commonly used loss function to measure the mean square error between the reconstructed signal and the real signal. L2 loss is sensitive to outliers, so it can effectively suppress large errors in many practical tasks. The smaller the L2 loss, the smaller the difference between the reconstructed signal and the original signal:

(15) $$L2\mathrm{Loss}=\frac{1}{N}\sum_{i=1}^{N}{\left(x_{\mathrm{true}}\left[i\right]-x_{\mathrm{pred}}\left[i\right]\right)}^{2}$$

L2 loss is often used to optimize objectives in model training, especially in regression and reconstruction tasks.

Figure 19g shows the sample size distribution of the denoised signal in different L2 loss intervals. The overall distribution shows a clear low-value concentration trend, indicating that most samples perform well in the root mean square error (L2 loss) metric and have small reconstruction errors.

The Pearson correlation coefficient (commonly used symbol *r* or ρ) is a standardized index to measure the degree of linear correlation between two continuous variables, which is defined as the ratio of the product of the sample covariance and the respective sample standard deviation, so the numerical range is strictly limited to [−1,1]; when r=1 indicates complete positive correlation, r=−1 indicates complete negative correlation, and r=0 indicates no linear relationship. The overall Pearson correlation coefficient is recorded as ρ, which is defined as the ratio of covariance and standard deviation of random variables X and Y:

(16) $$\rho_{X,Y}=\frac{\mathrm{Cov}(X,Y)}{\sigma_{X}\sigma_{Y}}=\frac{E[\left(X-\mu_{X}\right)\left(Y-\mu_{Y}\right)]}{\sigma_{X}\sigma_{Y}},$$

where $\mu_{X}=E\left[X\right],\mu_{Y}=E\left[Y\right],\sigma_{X},\sigma_{Y}$ are the standard deviation of *X* and *Y*, respectively. The corresponding sample Pearson correlation coefficient *r* (also known as the Pearson product–moment correlation coefficient) can be expressed as

(17) $$r=\frac{\sum_{i=1}^{n}\left(x_{i}-\bar{x}\right)\left(y_{i}-\bar{y}\right)}{\sqrt{\sum_{i=1}^{n}{\left(x_{i}-\bar{x}\right)}^{2}}\sqrt{\sum_{i=1}^{n}{\left(y_{i}-\bar{y}\right)}^{2}}},$$

where x and y are the sample means, respectively, the numerator is the unbiased estimation of the sample covariance, and the denominator is the product of the sample standard deviation.

Figure 19h shows the distribution of sample size of the denoised signal in different Pearson correlation coefficient (PCC) intervals. It can be observed that the PCC values are mainly concentrated in the high correlation interval, and the overall distribution tends to be close to the numerical range of 1, indicating that most samples maintain a high degree of linear correlation with the reference signal after denoising. This result indicates that the proposed method not only performs well in error metrics but also effectively preserves the global trend information of the signal.

Zero-lag cross-correlation (ZLCC) [32] is defined as the value of the cross-correlation function at lag zero, which is used to measure the similarity of two signals without timing offset. The non-normalized form of ZLCC is expressed as an integral in the continuous time domain and as a vector inner product in the discrete time domain, reflecting the synchronization relationship at the energy level of the original signal. If the signal is normalized by subtracting the mean value and dividing it by the standard deviation, the normalized zero-lag cross-correlation (ZNCC) is obtained. Its value range is strictly limited to [−1,1]: 1 means a fully positive correlation, −1 means a fully inverse correlation, and 0 means no linear correlation.

ZLCC is the value of the cross-correlation function at the lag τ=0 which is used to quantify the similarity between the signals *f* and *g* when there is no time shift alignment. For continuous signals f(t) and g(t),

(18) $$\mathrm{ZLCC}\left(f,g\right)=\left(f⋆g\right)\left(0\right)=\int_{-\infty }^{\infty }\bar{f\left(t\right)}g\left(t\right)dt,$$

where “⋆” indicates cross-correlation operation. If the signal is a real value, the complex conjugate symbol is omitted. For the discrete sequences $f\left[n\right]$,$g\left[n\right]$ with finite length (n=0,...,N−1),

(19) $$\mathrm{ZLCC}\left(f,g\right)=\sum_{n=0}^{N-1}f\left[n\right]g\left[n\right].$$

The sum is the inner product of two sequences in zero-lag alignment. Continuous form:

(20) $$\mathrm{ZLCC}\left(f,g\right)=\int_{-\infty }^{\infty }f\left(t\right)g\left(t\right)dt.$$

Discrete form:

(21) $$\mathrm{ZLCC}\left(f,g\right)=\sum_{n=0}^{N-1}f\left[n\right]g\left[n\right].$$

If the normalized zero-lag cross-correlation (ZNCC) is defined, subtract the respective mean values $\bar{f}\;$ and $\bar{g}\;$ and divide them by the standard deviations $\sigma_{f}$ and $\sigma_{g}$ to obtain

(22) $$\mathrm{ZNCC}\left(f,g\right)=\frac{\sum_{n=0}^{N-1}\left(f\left[n\right]-\bar{f}\right)\left(g\left[n\right]-\bar{g}\right)}{\sigma_{f}\sigma_{g}},$$

Its value range is [−1,1]. The range of non-normalized ZLCC values is not fixed. Its positive value indicates that the two signals are enhanced in the same phase under the current alignment, and its negative value indicates that the phase is opposite. The absolute size of the value is affected by the signal energy. The normalized ZNCC value falls strictly between [−1,1]: 1 means fully synchronous positive correlation, −1 means fully synchronous negative correlation, and 0 means no linear synchronization feature.

Figure 19i shows the sample size distribution of the denoised signal in different ZLCC value ranges. It can be seen that the ZLCC values are mostly concentrated in the high correlation region close to 1, indicating that without introducing time delay, the denoised signal and the reference signal are highly aligned in the time domain, with good synchronization and similarity. This distribution characteristic indicates that the proposed method can effectively preserve the phase information and overall morphology of the original signal while suppressing noise interference, demonstrating strong time-domain consistency recovery ability.

Table 5a,b list the average values of various evaluation indicators after applying denoising methods on the test set in order to comprehensively evaluate the denoising performance of the proposed method.

### 6.2. Signal Accuracy Improvement

If SNR is increased by ΔSNR dB, the reduction multiple of noise power is $\frac{P_{\mathrm{noise},\mathrm{old}}}{P_{\mathrm{noise},\mathrm{new}}}=10^{\frac{\Delta \mathrm{SNR}}{10}}$ Then the reduction percentage of noise power is calculated as $Noisereductionpercentage=\left(1-\frac{P_{\mathrm{noise},\mathrm{new}}}{P_{\mathrm{noise},\mathrm{old}}}\right)\times 100\%=\left(1-10^{-\frac{\Delta \mathrm{SNR}}{10}}\right)\times 100\%$ Similarly, if PSNR increases ΔPSNR dB, the reduction multiple of MSE is $\frac{{\mathrm{MSE}}_{\mathrm{old}}}{{\mathrm{MSE}}_{\mathrm{new}}}=10^{\frac{\Delta PSNR}{10}}$ Then the percentage of MSE reduction is calculated as

(23) $$\mathrm{MSE}\;\mathrm{reduction}\;\mathrm{percentage}=\left(1-10^{-\frac{\Delta PSNR}{10}}\right)\times 100\%$$

Table 6 shows the comparison of signal quality before and after denoising using the proposed denoising method, mainly including two indicators: signal-to-noise ratio (SNR) and peak signal-to-noise ratio (PSNR). As shown in the table, the SNR is increased by 13.90 dB, indicating a significant reduction in noise interference. According to the increase in SNR, the noise power is reduced by approximately 95.93%. At the same time, PSNR increased by 13.89 dB, and the corresponding mean square error (MSE) decreased by 95.92%. The above results indicate that the proposed method effectively suppresses noise while preserving the main features of the original signal, significantly improving signal quality.

**Figure A3:**
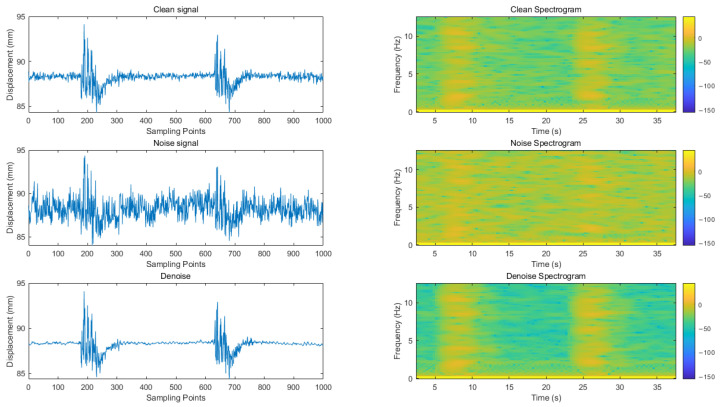
The waveform and time–frequency spectrum of a vibration signal with a frequency of 0.1 Hz and an amplitude of 0.81 mm.

**Figure A4:**
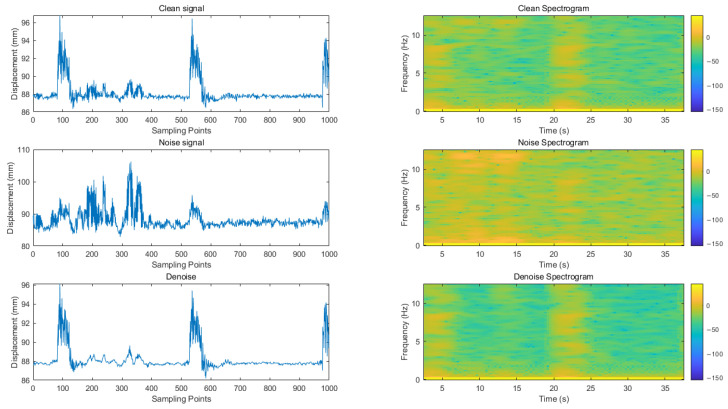
The waveform and time–frequency spectrum of a vibration signal with a frequency of 0.1 Hz and an amplitude of 0.75 mm.

**Figure A5:**
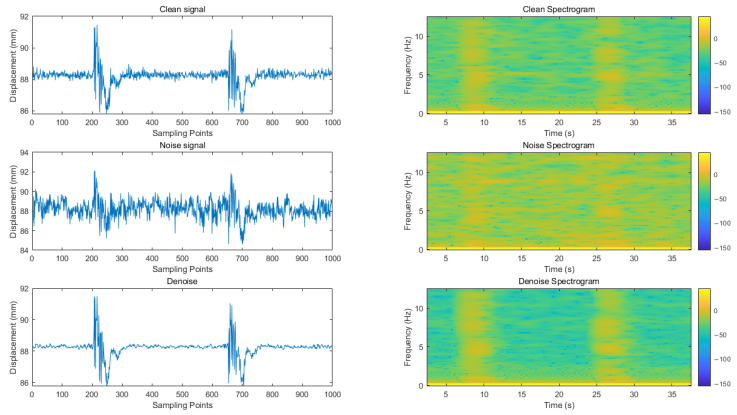
The waveform and time–frequency spectrum of a vibration signal with a frequency of 0.1 Hz and an amplitude of 0.43 mm.

**Figure A6:**
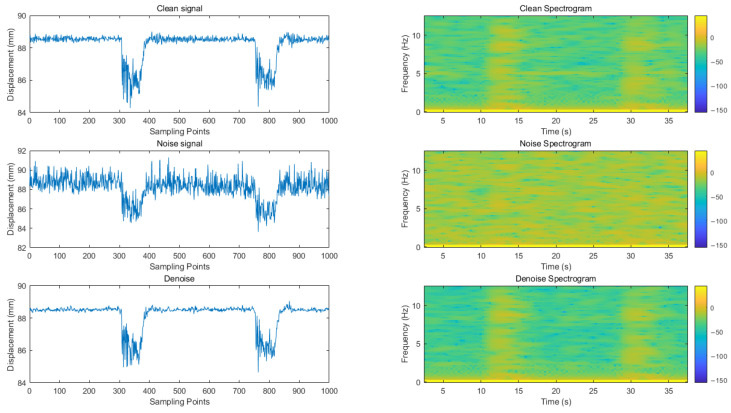
The waveform and time–frequency spectrum of a vibration signal with a frequency of 0.1 Hz and an amplitude of 0.31 mm.

**Figure A7:**
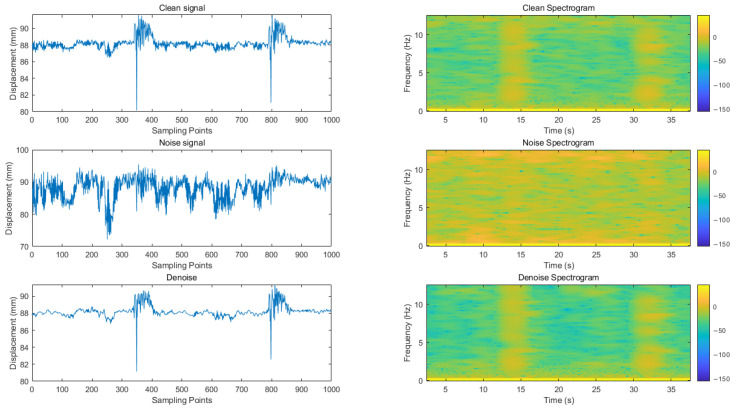
The waveform and time–frequency spectrum of a vibration signal with a frequency of 0.1 Hz and an amplitude of 0.93 mm.

**Figure A8:**
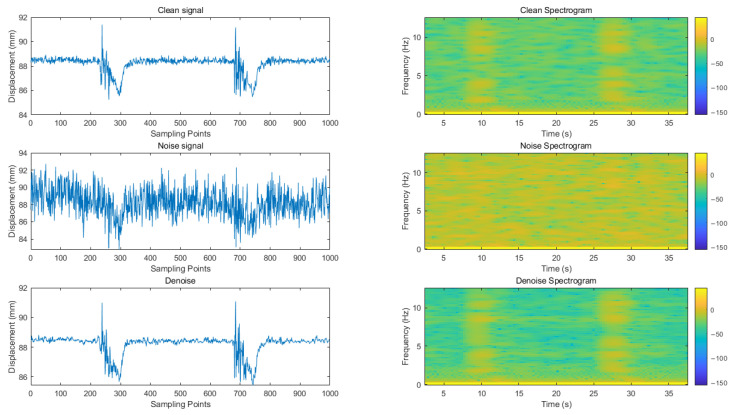
The waveform and time–frequency spectrum of a vibration signal with a frequency of 0.1 Hz and an amplitude of 0.50 mm.

In order to verify that an effective inference model can denoise vibration signals captured in real time outside the dataset, we select vibration signals with a frequency of 0.1 Hz and amplitudes of 0.81 mm, 0.75 mm, 0.43 mm, 0.31 mm, 0.93 mm, and 0.50 mm for denoising, and present waveform diagrams and time–frequency spectra as shown in Figure A3, Figure A4, Figure A5, Figure A6, Figure A7 and Figure A8 of Appendix A. The evaluation parameters after noise reduction are shown in Table 7a,b.

**Figure A9:**
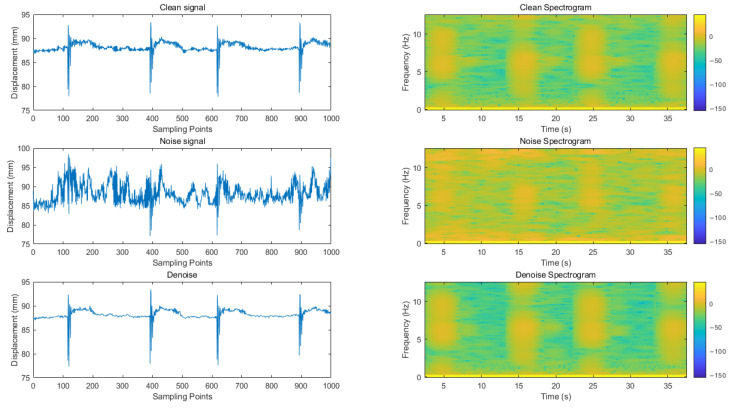
The waveform and time–frequency spectrum of a vibration signal with a frequency of 0.1 Hz and an amplitude of 1.5 mm.

**Figure A10:**
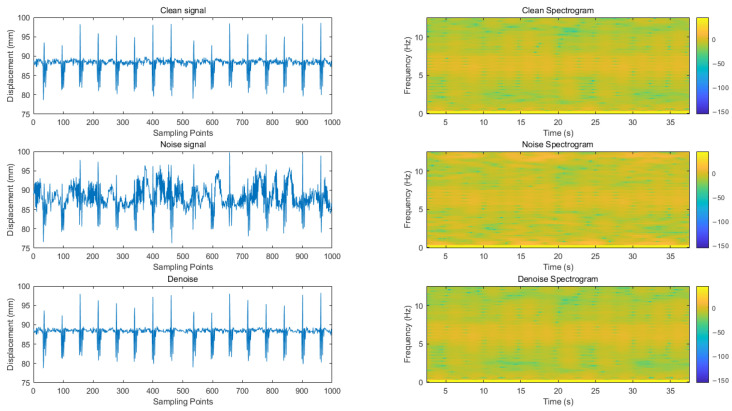
The waveform and time–frequency spectrum of a vibration signal with a frequency of 0.5 Hz and an amplitude of 1.5 mm.

**Figure A11:**
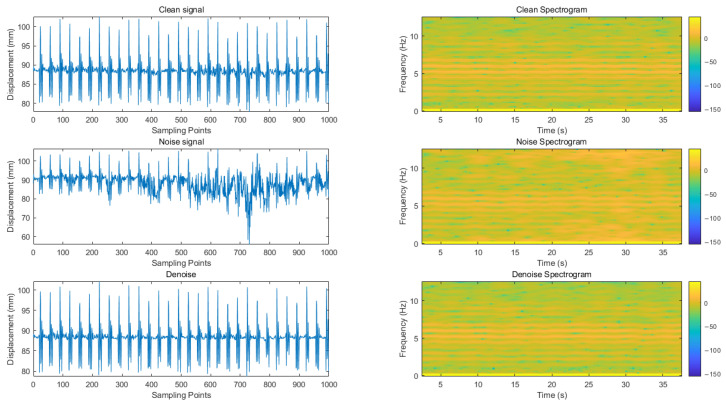
The waveform and time–frequency spectrum of a vibration signal with a frequency of 1 Hz and an amplitude of 1.5 mm.

At the same time, we also have good noise reduction effects on vibration signals with the same amplitude but different frequencies, fully demonstrating the robustness of the algorithm. The waveform and time–frequency spectra of vibration signals with an amplitude of 1.5 mm and frequencies of 0.1 Hz, 0.5 Hz, and 1 Hz are shown in Figure A9, Figure A10 and Figure A11 of Appendix A, and the detailed evaluation parameters after noise reduction are shown in Table 8a,b.

### 6.3. Comparative Experiments

In the comparative experiments, all models were trained and evaluated on the same vibration signal dataset. The proposed BiL-DCAE model was systematically compared with multiple advanced deep learning models, including RNN, GRU, Transformer, DCAE, LSTM-DAE, U-Net, and BiLSTM, as well as several classical denoising methods, including low-pass filtering, band-pass filtering, mean filtering, Savitzky–Golay filtering, Variational Mode Decomposition (VMD), wavelet transform, Wiener filtering, and Kalman filtering.

**Figure 20:**
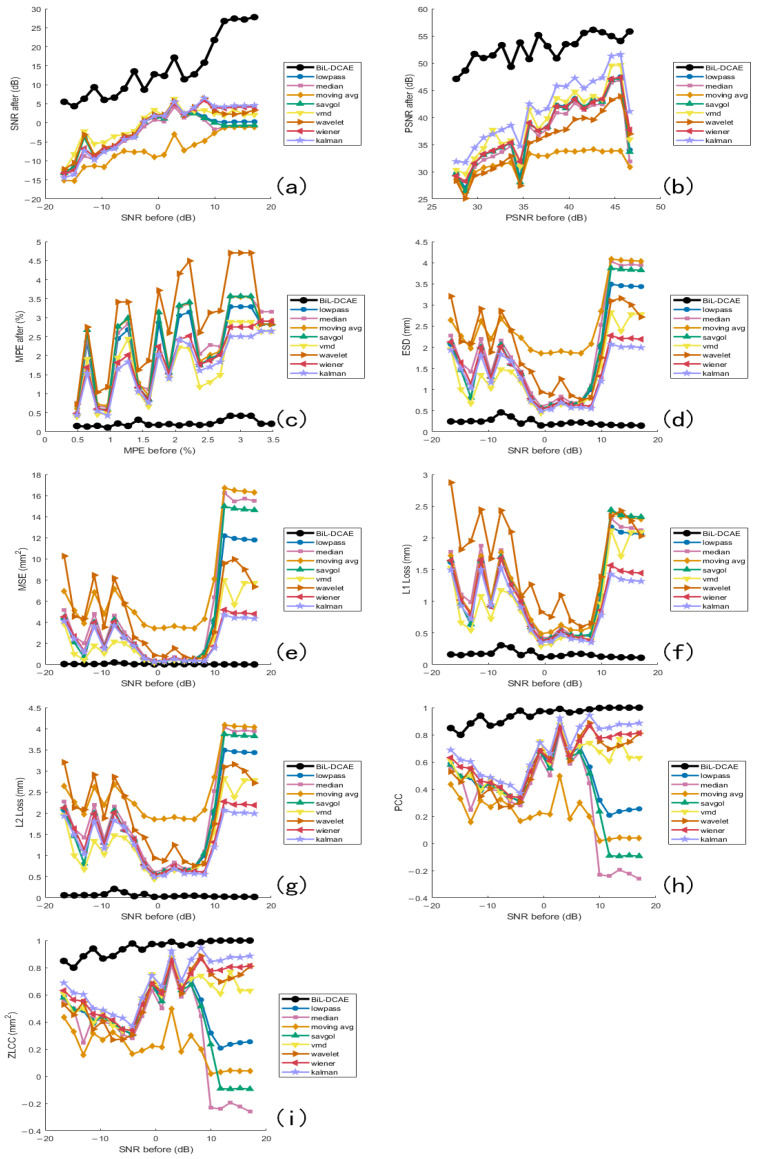
Comparative evaluation of BiL-DCAE and classical denoising methods across multiple metrics. Subfigures (**a**–**i**) present a comprehensive comparison between the proposed BiL-DCAE model and several classical denoising methods, including low-pass filtering, band-pass filtering, mean filtering, Savitzky–Golay filtering, Variational Mode Decomposition (VMD), wavelet transform, Wiener filtering, and Kalman filtering. The evaluation is conducted using multiple quantitative metrics: (**a**) SNR, (**b**) PSNR, (**c**) MPE, (**d**) ESD, (**e**) MSE, (**f**) L1 loss, (**g**) L2 loss, (**h**) PCC, and (**i**) ZLCC.

**Figure 21:**
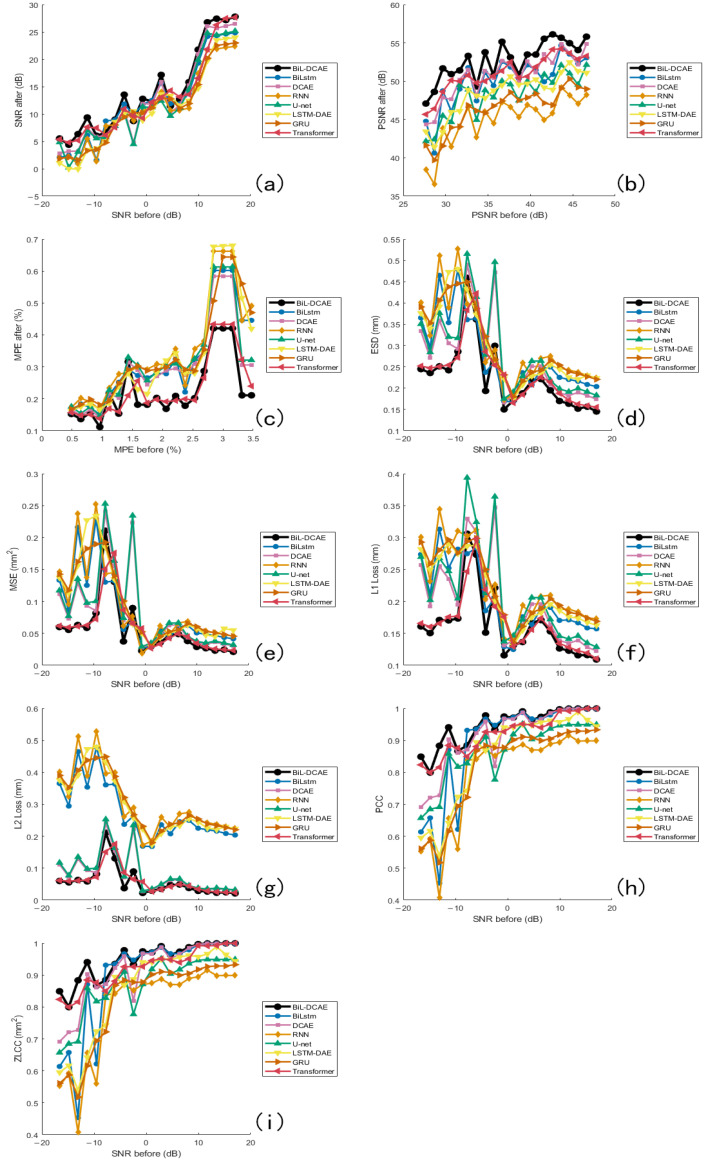
Comparative evaluation of BiL-DCAE and advanced deep learning models across multiple metrics. Subfigures (**a**–**i**) present a comprehensive comparison between the proposed BiL-DCAE model and advanced deep learning models, including RNN, GRU, Transformer, DCAE, LSTM-DAE, U-Net, and BiLSTM. The evaluation is conducted using multiple quantitative metrics: (**a**) SNR, (**b**) PSNR, (**c**) MPE, (**d**) ESD, (**e**) MSE, (**f**) L1 loss, (**g**) L2 loss, (**h**) PCC, and (**i**) ZLCC.

To improve clarity, the comparative results are presented in two figures: Figure 20 illustrates the comparison between BiL-DCAE and various classical denoising methods across multiple quantitative metrics, while Figure 21 provides a comprehensive comparison with advanced deep learning models.

For classical methods, BiL-DCAE overwhelmingly outperforms all baselines, with the Kalman filter ranking second but still showing a noticeable gap. Wiener filtering, VMD, and wavelet transform form the mid-performing group, while simple filters such as low-pass, Savitzky–Golay, mean, and moving average filters rank lowest, highlighting their limited capacity to handle non-stationary and complex noise.

In the deep learning group, BiL-DCAE consistently achieves the highest scores across all metrics, followed by Transformer-based models. Notably, Transformer approaches exhibit comparable SNR performance to BiL-DCAE in high-SNR regions but show greater instability across different noise levels, reflecting their sensitivity to data distribution and hyperparameter settings. While BiL-DCAE achieves overall higher PSNR values, Transformers slightly outperform in a few localized cases for ESD, MPE, and MSE, indicating their strong global modeling capability. However, BiL-DCAE demonstrates superior consistency across the entire range, particularly in peak preservation (MPE) and frequency-domain fidelity (ESD), which we attribute to its hybrid convolutional and bidirectional temporal modeling.

In L1 and L2 losses, BiL-DCAE maintains competitive performance, with L2 loss nearly matching Transformer in high-SNR regions, suggesting robust reconstruction under low-noise conditions. Correlation-based metrics (PCC and ZLCC) show that BiL-DCAE, Transformer, DCAE, and BiLSTM converge to similarly high values in high-SNR regions, indicating their shared ability to maintain overall waveform morphology. Overall ranking trends across metrics consistently place BiL-DCAE first, followed by Transformer, with DCAE, U-Net, and BiLSTM forming a secondary group, and RNN/GRU trailing due to their limited capacity for capturing long-term bidirectional dependencies.

These findings highlight that the hybrid architecture of BiL-DCAE, which combines convolutional layers for local time–frequency feature extraction and bidirectional LSTM modules for long-term temporal modeling, provides a significant advantage in suppressing noise while preserving essential signal characteristics.

## 7. Conclusions

On the basis of a study on seismic wave laser remote sensing detection based on Shack–Hartmann wavefront sensor, this article adopts a vibration signal detection system based on wavefront sensor. The transmitting end of the system utilizes the advantages of short laser wavelength, high detection sensitivity, and high measurement resolution as a carrier for vibration signal acquisition; the receiving end utilizes the small size, high precision, high resolution, and high sensitivity of the Shack–Hartmann wavefront sensor to fully capture vibration signals with information. This article is based on the existing system and conducts experimental research on single-point vibration. According to the basic principle of wavefront sensors, we analyze and verify that when the vibration source is excited, the centroid offset of the wavefront sensor spot is proportional to the amplitude, and calculate the proportionality coefficient. At the same time, this study proposes a vibration signal denoising method based on the BiL-DCAE neural network architecture. By collecting a large number of vibration signals received by wavefront sensors in different experimental environments, a real signal dataset is constructed for feature learning, and the denoising effect is systematically analyzed through multiple objective evaluation indicators. The experimental results show that the denoised signal has achieved significant improvements in indicators such as signal-to-noise ratio (SNR), mean square error (MSE), and zero-lag cross-correlation (ZLCC). Compared with traditional denoising methods, BiL-DCAE can more effectively suppress noise while maintaining the key structural information of the signal.

In addition, time–frequency analysis further verified the effectiveness of the method, and the denoised signal not only approximates the original clean signal in the time domain but also preserves the main frequency components of the original signal well in the frequency domain. At the same time, this article also uses the trained model for inference denoising outside the dataset, while maintaining excellent denoising performance. This method can effectively denoise signals of different amplitudes and frequencies, indicating that the model has strong robustness and generalization ability. These results indicate that BiL-DCAE has superior noise reduction performance in vibration signal processing.

This article conducts experiments on low-frequency vibration and small amplitude vibration, observes the various characteristics of the system, and conducts single point vibration detection and signal denoising of vibration signals. The collected noisy vibration signals are denoised to significantly improve the quality and analyzability of the signals, thereby more accurately extracting vibration feature information and improving the reliability and effectiveness of subsequent signal analysis. This capability enables the system to maintain the high-precision detection of small vibrations in complex environments, providing a solid data foundation for pattern recognition, state monitoring, and anomaly detection of vibration signals. At the same time, the denoised signal is closer to the real vibration signal in both the time and frequency domains, making it capable of meeting the requirements of precision measurement and remote detection in the later stage of the system.

In summary, the scientific value of this work lies in two main aspects. First, it experimentally validates the proportional relationship between the centroid offset of the Shack–Hartmann wavefront sensor spot and the vibration amplitude, providing solid experimental evidence for the theoretical foundation of laser-based seismic vibration sensing. Second, it constructs and applies a BiL-DCAE neural network specifically for vibration signal denoising, trained on a self-collected real vibration signal dataset. Through systematic comparison with multiple traditional denoising methods and advanced deep learning models, the proposed BiL-DCAE achieves the best performance across diverse quantitative metrics. These contributions advance high-precision seismic vibration detection methodology and offer a robust, generalizable signal processing solution for broader applications in vibration monitoring and geophysical exploration.

Looking ahead, the system can be further expanded to more complex vibration environments, such as high sampling density and large viewing angle detection, as well as applications such as long-distance laser vibration measurement. By combining higher-precision OPA phased array radar, the system’s ability to detect weak vibration signals can be further improved, making it applicable to a wider range of scenarios.

In addition, in order to enhance the intelligence level of the system, unsupervised learning methods can be introduced in the future to enable the model to train solely on noisy signals without providing clean signals, thus achieving more flexible adaptive noise reduction. This will help the system to break free from dependence on prior data in practical applications and achieve real-time, online vibration signal denoising and analysis.

Furthermore, in future work, we plan to extend our approach beyond signal denoising toward a deeper analysis of the dynamic nonlinearity of the medium. Specifically, the high-quality vibration signals obtained in this study could facilitate the phase-resolved decomposition of the nonlinear response into its reactive (energy-storing) and dissipative (energy-dissipating) components under pulsating loads. This extension would provide a more comprehensive understanding of the medium’s nonlinear behavior, complementing the current work.

Moreover, these high-quality signals can serve as valuable inputs for advanced geophysical workflows in the future. In particular, we plan to integrate the denoised wavefront-derived vibration data into seismic inversion frameworks, enabling the reconstruction of subsurface elastic parameters and structural features. This extension will bridge our current work with practical geological and geophysical exploration tasks, such as fault zone characterization, site response assessment, and seismic hazard evaluation, ultimately supporting informed and optimized engineering decision-making.

At the same time, the system also has important application value in the development of portability and integration by developing vibration monitoring equipment that is lightweight, efficient, and low-power. Through multi-source data fusion and intelligent signal processing, the robustness and adaptability of the system are further improved, providing more efficient and accurate solutions for precision vibration measurement and vibration signal analysis in complex environments.

## Figures and Tables

**Table 1:** Parameters of the WFS-20-5C Shack–Hartmann wavefront sensor.

Wavelength Range/nm	300–1100
Lenslet Pitch/μm	150
Lens Diameter/μm	146
Wavefront Accuracy	λ/30 rms @ 633 nm
Wavefront Sensitivity	λ/100 rms @ 633 nm
Wavefront Dynamic Range	>100λ@ 633 nm
Pixel size/μm	5 × 5
Framerate/($f•s^{-1}$)	23–880

**Table 2:** Composition of dataset.

	Clean_Test	Clean_Train	Noise_Test	Noise_Train
number	5150	38,522	5150	38,522

**Table 3:** The server parameters.

Parameter Name	Parameter Value
operating system	Ubuntu 18.04.5 LTS
system memory	94.3GiB
CPU	Intel® Xeon(R) CPU E5-2690 v3 @ 2.60GHz × 48
GPU	GeForce RTX 3090

**Table 4:** Optimal hyperparameter configuration obtained through Hyperopt.

Parameter	Search Range	Optimal Value
Convolutional Layers	3–5	6
Kernel Sizes	{3, 4, 5, 7}	5/5/4/3/3/3
BiLSTM Units (per layer)	64–256	100
Dropout Rate	0.2–0.5	0.2–0.3
Learning Rate	1 × $10^{-4}$–1 × $10^{-2}$	1 × $10^{-3}$

**Table 5:** Average values of evaluation indexes in test set after denoising.

a Evaluation results (SNR, PSNR, and MPE)						
	SNR Before (dB)	SNR After (dB)	PSNR Before (dB)	PSNR After (dB)	MPE Before (%)	MPE After (%)
Average	−4.52	9.38	38.08	51.97	1.18	0.20
b Evaluation results (ESD, MSE, L1 Loss, L2 Loss, PCC, ZLCC)						
	ESD (mm)	MSE (${\mathrm{mm}}^{2}$)	L1 Loss (mm)	L2 Loss (mm)	PCC	ZLCC (${\mathrm{mm}}^{2}$)
Average	0.24336	0.06557	0.1796	0.0656	0.9358	0.9358

**Table 6:** Comparison of SNR and PSNR before and after noise reduction.

	Before Noise Reduction	After Noise Reduction
SNR	−4.52 dB	9.38 dB
PSNR	38.08 dB	51.97 dB

**Table 7:** The evaluation results of vibration signals with frequencies of 0.1 Hz and amplitudes of 0.81, 0.75, 0.43, 0.31, 0.93, and 0.50 mm.

a Evaluation results (SNR, PSNR, and MPE)						
Signal amplitude (mm)	SNR Before (dB)	SNR After (dB)	PSNR Before (dB)	PSNR After (dB)	MPE Before (%)	MPE After (%)
0.81	−10.0474	10.15186	31.73602	51.92444	2.10506	0.206157
0.75	−5.75101	9.507248	36.67054	51.92303	1.245596	0.169698
0.43	1.918836	14.98143	42.25753	55.31398	0.623677	0.135307
0.31	−1.65367	9.149556	42.79611	53.59887	0.600475	0.164295
0.93	−5.99629	14.07087	31.14856	51.21499	1.788265	0.216891
0.50	−0.75442	11.06646	40.52655	52.33497	0.81396	0.200164
b Evaluation results (ESD, MSE, L1 Loss, L2 Loss, PCC, ZLCC)						
Signal amplitude (mm)	ESD (mm)	MSE (${\mathrm{mm}}^{2}$)	L1 Loss (mm)	L2 Loss (mm)	PCC	ZLCC (${\mathrm{mm}}^{2}$)
0.81	0.231984	0.053816	0.181783	0.053816	0.95829	0.95829
0.75	0.231599	0.053638	0.149503	0.053638	0.980275	0.980275
0.43	0.152653	0.023303	0.119103	0.023303	0.940619	0.940619
0.31	0.191128	0.03653	0.14486	0.03653	0.970308	0.970308
0.93	0.266239	0.070883	0.191729	0.070883	0.932291	0.932291
0.50	0.227504	0.051758	0.176565	0.051758	0.923719	0.923719

**Table 8:** Evaluation index results of vibration signals with an amplitude of 1.5 mm and frequencies of 0.1 Hz, 0.5 Hz, and 1 Hz.

a Evaluation results (SNR, PSNR, and MPE)						
Signal frequency	SNR Before (dB)	SNR After (dB)	PSNR Before (dB)	PSNR After (dB)	MPE Before (%)	MPE After (%)
0.1 Hz	−5.39177	12.43008	32.26501	50.08466	2.085175	0.264146
0.5 Hz	−0.87359	16.94872	32.56446	50.34434	2.158481	0.263009
1 Hz	−0.79376	15.50205	28.35483	44.62778	3.549271	0.465563
b Evaluation results (ESD, MSE, L1 Loss, L2 Loss, PCC, ZLCC)						
Signal frequency	ESD (mm)	MSE (${\mathrm{mm}}^{2}$)	L1 Loss (mm)	L2 Loss (mm)	PCC	ZLCC (${\mathrm{mm}}^{2}$)
0.1 Hz	0.292475	0.085542	0.232764	0.085542	0.971083	0.971083
0.5 Hz	0.299556	0.089734	0.232107	0.089734	0.989992	0.989992
1 Hz	0.602324	0.362794	0.409736	0.362794	0.986955	0.986955

**Figure A1:**
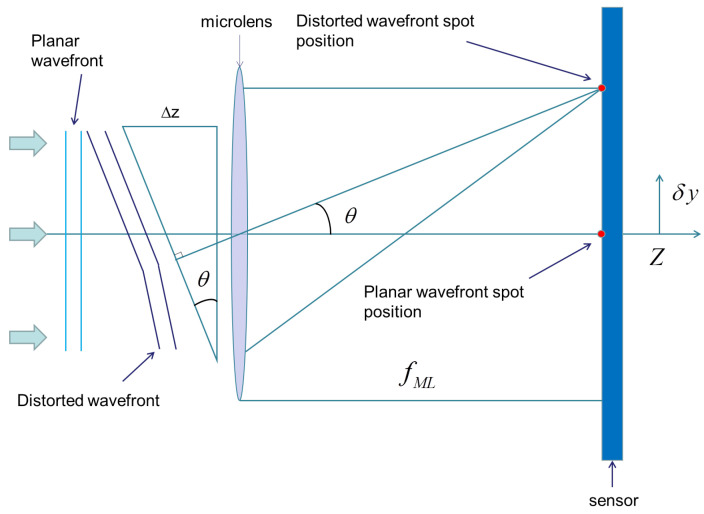
Schematic diagram of single microlens imaging principle.

## Data Availability

The data that support the findings of this study are available from the corresponding author upon reasonable request.

## Appendix A

Rodier pointed out that the microlens array in a Shack–Hartmann wavefront sensor (SHWFS) can be regarded as a phase grating, and the entire SHWFS can be treated as a phase grating interferometer. The phase grating function G(x,y), representing the distribution of the entire wavefront over the microlens array, is a biperiodic function expressed as

(A1) $$G\left(x,y\right)=\frac{1}{p^{2}}\sum_{n=-\infty }^{+\infty }\sum_{m=-\infty }^{+\infty }C_{n,m}\left(t\right)e^{\frac{2i\pi }{p}\left(nx+my\right)}$$

where *P* is the spacing between microlenses, *t* denotes time, and $C_{n,m}\left(t\right)$ is the coefficient corresponding to each spatial frequency. These coefficients can be interpreted as the Fourier transform of the complex pupil function of a single microlens:

(A2) $$C_{n,m}=\mathrm{FT}{\left[\prod_{p,p}\left(x,y\right)e^{\frac{i\pi \left(x^{2}+y^{2}\right)}{\lambda f_{\mathrm{ML}}}}\right]}_{\frac{n}{p},\frac{m}{p}}$$

where λ is the laser wavelength, $f_{\mathrm{ML}}$ is the focal length of the microlens, and $\Pi_{p,p}\left(x,y\right)$ denotes the aperture function of the microlens. For simplicity, we assume a rectangular microlens aperture and apply the paraxial (small-angle) approximation, which are reasonable simplifications in SHWFS modeling. For practical analysis, $C_{n,m}$ can also be expressed as

(A3) $$C_{n,m}=\psi_{\mathrm{ML}}\left(\frac{n}{p},\frac{m}{p}\right)=\left[\frac{\sin\left(n\pi \right)}{n\pi }\frac{\sin\left(m\pi \right)}{m\pi }\right]\times e^{i\pi \lambda f_{\mathrm{ML}}\left(u^{2}+\upsilon^{2}\right)}$$

where u,v is the displacement of the light spot in the focal plane of the microlens. Referring to the imaging principle of the distorted wavefront containing vibration information (see Figure A1), take the *y*-direction as an example: the object vibration amplitude is $\Delta Z_{y}$, and the corresponding displacement of the spot on the focal plane is $\delta_{y}$. From simple geometric relations,

(A4) $$\tan\alpha =\frac{\Delta Z_{y}}{\Delta y}=\frac{\delta y}{f_{\mathrm{ML}}}$$

(A5) $$\tan\beta =\frac{\Delta Z_{x}}{\Delta x}=\frac{\delta x}{f_{\mathrm{ML}}}$$

Substituting these geometric relationships into the Fourier coefficient expression gives the final imaging equation for the distorted wavefront:

(A6) $$C_{n,m}=\left[\frac{\sin\left(\pi n+\frac{x^{\prime }}{tg\alpha }\right)}{n\pi }\frac{\sin\left(\pi m+\frac{y^{\prime }}{tg\beta }\right)}{m\pi }\right]\times e^{i\pi \lambda f_{\mathrm{ML}}\left[{\left(\pi n+\frac{x^{\prime }}{tg\alpha }\right)}^{2}+{\left(\pi m+\frac{y^{\prime }}{tg\beta }\right)}^{2}\right]}$$

where $\left(x^{\prime },y^{\prime }\right)$ is the coordinate of any point on the object surface. This indicates that the displacement of the light spot encodes the distortion of the laser wavefront, i.e., the phase information, and that this distortion originates from the object’s vibration. Thus, by measuring the spot displacement, the changes in the wavefront phase and the vibration information that caused them can be obtained.
